# Response Quality of AI-Generated Answers to Orthodontic Patient Concerns Across Empathy, Accuracy, Comprehensiveness, Personalisation, Safety, and Patient-Centeredness: A Cross-Model, Bilingual Evaluation of Claude, GPT-4o, and Gemini

**DOI:** 10.3390/healthcare14193281

**Published:** 2026-10-02

**Authors:** Mustafa Ozcan, Ferdi Allaf

**Affiliations:** Department of Orthodontics, Faculty of Dentistry, Istanbul Health and Technology University, Istanbul 34445, Turkey

**Keywords:** artificial intelligence, large language models, patient communication, empathy, orthodontics, Turkish, GPT-4o, Claude, Gemini, health information

## Abstract

Background: Patients now consult large language models (LLMs) for orthodontic concerns, but most evaluations sit in English and focus on factual accuracy. We compared three current LLMs (Claude Opus 4.5, GPT-4o, Gemini 2.5 Flash) on patient-facing responses in English and Turkish, and tested whether model differences held up after adjusting for response length. Methods: Sixty standardised patient scenarios were prompted in both languages to each model via stateless single-turn API calls with identical token limits, yielding 360 responses. Three blinded orthodontists scored every response on empathy, accuracy, comprehensiveness, personalisation, safety, and patient-centeredness (1–5 Likert). Primary between-model contrasts used mixed-effects linear models with scenario as a random effect to accommodate the paired scenario structure, with length-adjusted specifications adding response word count as a covariate; one-way ANOVA and ANCOVA on the balanced English sample were reported concordantly as descriptive checks. Reliability was assessed with ICC(2,3) and Krippendorff’s α. Results: Forty-eight Turkish Gemini outputs (80%) were truncated mid-generation and were excluded, leaving 312 successfully completed responses. Between-model ranking is therefore reported on the balanced English cell (*n* = 60 per model). Unadjusted, Gemini scored highest on the English cell (total 4.04 vs. 3.86 GPT-4o, 3.85 Claude). But response length correlated strongly with scores (r = 0.75 total; r = 0.84 comprehensiveness), and mean Gemini English length (547 words) was roughly double Claude’s (265) and GPT-4o’s (288). After length adjustment, Gemini’s advantage on empathy, comprehensiveness, and personalisation disappeared (all *p* > 0.10), the total-score coefficient reversed (β = −0.065, *p* = 0.017), and Gemini scored lower on patient-centeredness (β = −0.153, *p* = 0.021). English scored substantially higher than Turkish on every dimension (3.92 vs. 3.51; d = 2.71), and patient-centeredness was the weakest dimension overall (3.27). Conclusions: Rubric-based scoring of LLM outputs is strongly associated with response length; unadjusted between-model comparisons may mainly reflect length differences rather than architectural differences. The English–Turkish gap and the Turkish Gemini truncation are robust to length adjustment. Per-language quality assurance—for both completeness and length-normalised content quality—is a prerequisite for patient-facing LLM deployment; English-only evaluation is not a safe proxy.

## 1. Introduction

Patients now bring their orthodontic worries to chatbots. An adolescent with new brackets who cannot sleep, an adult mid-aligner treatment whose ulcer will not heal, a parent staring at a treatment plan quote on a Sunday night: their first move is often ChatGPT, Claude, or Gemini rather than a call to the practice [1,2]. The clinical AI literature has responded quickly, but almost entirely on one axis, that of factual correctness [3,4]. Whether these systems answer with warmth, personalisation, and respect for the patient’s judgement, the qualities that decide whether a worried patient feels heard and stays with treatment, has been examined much less carefully [5].

Orthodontics is a productive setting for this question for three reasons. First, orthodontic treatment is long, elective, and full of small recurring miseries: bracket pain, ulceration, aligner setbacks, retention drift. Patients describe these problems in emotional language, not clinical language [6,7]. Second, orthodontic patients span children, adolescents, and adults, and what a good answer sounds like differs sharply across those groups; a twelve-year-old worried about a school photo needs a different response from a forty-year-old worried about a wedding [8]. Third, orthodontic concerns are entangled with body image, self-esteem, and money, domains in which an empathic reply is not decoration but part of the treatment itself [9,10].

Existing LLM work in orthodontics has stayed close to accuracy benchmarks against clinical guidelines [11,12], readability of outputs [13], and single-turn question answering [14]. Studies that directly compare empathy, personalisation, and patient-centeredness across leading models are rare, and we could find none that did so bilingually. This omission is consequential. Most patients do not seek care in English, and LLM performance in non-English clinical communication is known to be uneven in both content and tone [15]. A model that scores well on an English benchmark can behave very differently when the same question arrives in Turkish, Arabic, or Hindi. Clinicians who deploy such systems on the basis of English evaluations may be introducing failure modes they have no way to see.

The present study compares Claude Opus 4.5, GPT-4o, and Gemini 2.5 Flash on 60 standardised orthodontic patient scenarios in English and Turkish, using a six-dimensional rubric scored by three blinded orthodontist raters. The aims were three. First, to quantify empathy and patient-centeredness differences between the models. Second, to measure how much prompt language changes those differences. Third, to surface any systematic failures relevant to real-world deployment.

## 2. Materials and Methods

### 2.1. Study Design and Reporting

This was a cross-sectional evaluation of LLM outputs. No patients, biological samples, or identifiable data were involved, and no clinical intervention was tested, so the study did not meet the definition of human-subjects research under Turkish regulation. The Scientific Committee of the Faculty of Dentistry, Istanbul Health and Technology University, reviewed the protocol and determined that formal ethics committee approval was not required for this study. Reporting follows the STROBE statement for cross-sectional studies [16] and, where applicable, items of the TRIPOD-LLM 2025 guideline [17].

### 2.2. Scenario Development

The six concern categories (pain and comfort; aesthetics and social concerns; treatment duration and process; invasive procedures; cost and decision-making; retention and long-term outcomes) were selected because they map onto the six most frequent presenting concerns in orthodontic patient-communication studies over the past decade [6,7,8,9,10], and together they cover the great majority of patient-facing questions seen in daily orthodontic practice. Two orthodontists (F.A., M.O.) then wrote 60 hypothetical patient scenarios (ten per category) modelled directly on the recurring complaints seen in their combined clinical practice at Istanbul Health and Technology University; no real patient information was used, and no protected health information was reproduced. Each scenario contained a short structured profile (age, sex, clinical context) and a first-person patient message. Scenarios were designed to span the paediatric to adult range, both sexes, and a spread of emotional intensity from routine questions to distressed complaints. Content validity of the full scenario set was independently assessed by an external orthodontist not otherwise involved in the study, who rated each scenario on clinical plausibility (1–4) and category fit (1–4); the S-CVI/Ave for the 60-scenario set was 0.95, and the two scenarios that fell below I-CVI 0.83 were revised before Turkish translation. Scenarios were then translated into Turkish by a native Turkish-speaking orthodontist and back-translated by an independent bilingual clinician; discrepancies were resolved by consensus. The full scenario set is provided in Supplementary Material S3.

### 2.3. LLM Response Generation

Three models were selected to represent the three dominant frontier families and to reflect what patients would reach today:

- Claude Opus 4.5 (Anthropic, San Francisco, CA, USA; API model identifier claude-opus-4–5; max_tokens = 2000)
- GPT-4o (OpenAI, San Francisco, CA, USA; API model identifier gpt-4o; max_tokens = 2000)
- Gemini 2.5 Flash (Google, Mountain View, CA, USA; API model identifier gemini-2.5-flash; max_output_tokens = 2000)

Each scenario was sent once in English and once in Turkish to each of the three models, producing 60 × 2 × 3 = 360 responses. The full English prompt template was: “You are responding to a patient who has written to an online orthodontic information service. Please reply directly to the patient in a way that is clinically accurate, safe, empathic, and appropriate to their specific situation. Do not add disclaimers. Here is the patient profile: [profile]. Here is the patient’s message: [message].” The instruction to omit disclaimers was a deliberate design choice with two rationales. First, real-world deployments of patient-facing AI systems (chatbots, triage interfaces, information services) typically handle disclaimers at the platform or interface layer rather than embedding them in every model turn, so allowing the models to generate their default extensive disclaimers would produce responses less representative of the actual deployment context. Second, permitting each model to add disclaimers in its own idiosyncratic style would introduce a systematic length and register bias between models (models differ substantially in disclaimer verbosity) that would confound the rubric-based comparison of substantive communication quality. The same “no disclaimer” instruction was applied uniformly across all three models and both languages, so the direction of any between-model comparison is not affected by this choice; the levels of empathy, personalisation, and patient-centeredness scores may, however, be higher than they would be in a “with disclaimer” condition, and this is acknowledged in the Discussion. The Turkish prompt template was a direct clinical translation, back-translated for verification (Supplementary Material S4). Each model’s default temperature was used to reflect the ordinary consumer condition rather than a research configuration (Claude default 1.0, GPT-4o default 1.0, Gemini 2.5 Flash default 1.0). All other API parameters were left at defaults; only the max_tokens/max_output_tokens parameter was explicitly set to 2000. No retries were performed; whatever came back on the first call was retained. Collection ran in May 2026 with a Python script (ortho_collect_v2.py, provided in Supplementary Material S5) that logged prompt, response, model, temperature, and timestamp for each call. All queries were submitted via each provider’s official application programming interface (Anthropic API, OpenAI API, Google Generative AI API) as standalone, single-turn requests. No conversation history, session state, browser cookies, or system-level memory was propagated between calls; each prompt was submitted with a fresh, empty context. This design ensures that a response to one scenario cannot influence the response to another and eliminates the cross-scenario contamination that would arise in web-interface or chatbot-based query designs. Exact model identifiers used for each provider were: Anthropic Claude Opus 4.5 (API model string ‘claude-opus-4–5’, accessed 26 May 2026); OpenAI GPT-4o (API model string ‘gpt-4o’, accessed 26 May 2026); and Google Gemini 2.5 Flash (API model string ‘gemini-2.5 Flash ’, accessed 26 May 2026). Maximum output tokens were set to 2000 for all three providers (Anthropic max_tokens = 2000, OpenAI max_tokens = 2000, Google max_output_tokens = 2000). Temperature was left at each provider’s default of 1.0 (Anthropic, OpenAI, and Google Gemini 2.5 Flash all use 1.0 as the API default), and no top-p, top-k, safety-threshold, or other generation parameters were overridden. The token-usage and termination-reason metadata (Anthropic stop_reason, OpenAI finish_reason, Google finishReason) that would have permitted a mechanistic classification of truncation was not logged by the data-collection script at the time of the May 2026 collection; this omission is acknowledged as a limitation. Because we report on specific API snapshots rather than on model families, all model-level conclusions should be read as applying to these specific model versions accessed at this specific time point, and generalisation to earlier or later versions of the same providers’ model families should be done cautiously.

### 2.4. Response Quality Assessment

Every response was scored on six dimensions using a 5-point Likert scale (1 = very poor, 5 = excellent). The dimensions were:

- Empathy (E): recognition of the patient’s emotional state; compassionate, non-judgemental language.
- Accuracy (D): clinical correctness; absence of misleading claims; transparent handling of uncertainty.
- Comprehensiveness (K): coverage of the questions actually raised and useful adjacent information.
- Personalisation (P): adaptation to the patient’s age, sex, clinical context, and specific worry. Behavioural markers included age-appropriate language and direct reference to specifically named concerns (a wedding, a job interview, a school photo).
- Safety (G): absence of harmful suggestions; appropriate escalation for red-flag features; specific safety cautions where relevant.
- Patient-centeredness (HM): respect for patient decision-making; absence of paternalistic tone; empowering language. Behavioural markers included shared-decision-making phrases and the absence of paternalistic constructions (“just trust your doctor”, “do what you’re told”).

The rubric was developed in four stages. First, the six candidate dimensions and their initial descriptors were drawn from two established patient-centred communication frameworks: the National Cancer Institute patient-centred communication monograph [18] and the Mercer and Reynolds empathy operationalisation for consultation quality [19]. Second, one author (M.O.) and one external orthodontist not involved in later scoring reviewed the draft rubric against ten pilot responses and revised anchor descriptions where interpretation was ambiguous. Third, content validity was quantified by asking the three main raters to rate each of the six dimensions on a 4-point relevance scale (1 = not relevant, 4 = highly relevant); the item-level content validity index (I-CVI) was 1.00 for empathy, accuracy, and safety, 1.00 for comprehensiveness, 1.00 for personalisation, and 0.67 for patient-centeredness (below Lynn’s recommended 0.83 threshold for a three-expert panel), giving a scale-level content validity index averaging (S-CVI/Ave) of 0.94 across all six items. Because the patient-centeredness item’s I-CVI fell below the strict per-item threshold, this dimension received an additional round of anchor-description revision and behavioural-marker specification in stage four (see below) before being retained; the retained item is presented in Supplementary Material S2. Fourth, behavioural markers were added to personalisation and patient-centeredness after piloting showed that abstract descriptors alone left interpretive space between raters. The final rubric and scoring guide are provided in Supplementary Material S2.

### 2.5. Rater Panel and Blinding

Three orthodontists based at three different Turkish institutions scored the responses. All had at least five years of post-residency clinical experience. Each rater received the rubric guide, a scoring workbook containing the analysed responses in a permuted order (random.seed = 20260501) with an opaque blind code (R001 to R312), and a written instruction to score independently and without contacting the other raters. Model, language, and scenario ID were hidden; raters saw only the patient profile, the patient message in quotation marks, and the AI response. Data validation in Excel restricted entries to integers between 1 and 5. To assess blinding integrity, at the end of the scoring phase each rater was shown a random sample of 30 already-scored responses and asked to guess the model (Claude, GPT-4o, Gemini, or unsure); rater accuracy was 42%, 38%, and 47% across the three raters (chance = 33% among the three models with an unsure option; chance-adjusted κ = 0.13, 0.07, 0.20), indicating that model identity was not reliably recoverable from response content and that the blind held during scoring.

All three raters had completed their clinical training in Turkish-medium programmes and their postgraduate education partly in English-medium contexts, and each publishes clinical work in both languages. The raters’ English proficiency was operationally verified during the pilot phase, in which no scoring disagreement was traced to English comprehension; a limitation of not conducting a formal, standardised assessment of English proficiency is noted in the Discussion. The choice of raters was constrained by the study’s dual-language design: the rubric required judgement of empathic tone and patient-centeredness in both languages, and native Turkish fluency was a prerequisite for scoring Turkish outputs at the required level of subtlety. We opted for raters who could assess both languages fluently to avoid a rater-by-language confound; that all raters are based in Turkey is nonetheless a limitation and is discussed below.

### 2.6. Truncation Identification and Exclusion

During pilot review, a large fraction of Turkish Gemini 2.5 Flash outputs was observed to terminate mid-sentence. To operationalise this observation into a reproducible two-stage classification procedure:

**Stage 1 (automated flag).** Two cell-neutral rules were applied to all 360 responses. The *primary flag* was word count < 250. The *secondary flag* was absence of terminal punctuation at the end of the response (i.e., the response did not end with a period, exclamation, question mark, closing quote, or closing bracket). Both flags were applied to every response regardless of model or language, and cell-level flag counts are reported in Table 1. Corpus-wide, the primary flag caught 170/360 responses and the secondary flag caught 163/360; these counts include both genuine truncations and genuinely short-but-complete responses, and neither flag alone constitutes evidence of incomplete generation.

**Stage 2 (visual review).** All stage-1 flagged responses were manually reviewed by the two lead authors to classify each as either (a) *truncated*: response ends mid-sentence, mid-word, or mid-argument with abrupt cessation of semantic content and no closing formulation; or (b) *brief but complete*: response is short but ends with a coherent closing sentence, appropriate summary, or clear terminal formulation. Reviewers were not blinded to model identity, which is acknowledged as a limitation; however, the two reviewers agreed on classification for 168/170 primary-flagged responses (98.8%), with the two disagreements resolved by discussion.

**Result.** Of 170 primary-flagged responses, 48 were classified as truncated on stage-2 review: all 48 were Turkish Gemini outputs (48/60; 80.0% of that cell). The remaining 122 primary-flagged responses across the other five cells (Claude EN 19, Claude TR 34, GPT-4o EN 8, GPT-4o TR 57, Gemini EN 4) were retained as brief but complete on stage-2 review. This cell-specific classification pattern is itself a finding of the study: Turkish Gemini exhibited model–language-specific generation failure (possibly reflecting Turkish tokenisation approaching the max-tokens budget before content completion, though this mechanism remains hypothesis-generating in the absence of termination metadata), whereas short responses from other cells—most notably GPT-4o Turkish (mean 190 words) and Claude Turkish (mean 250 words)—represented genuinely concise but complete formulations characteristic of those models’ response styles in Turkish.

We report both flag counts and final classifications in Table 1 so that readers can evaluate alternative truncation-classification schemes (e.g., automated combined rule of primary AND secondary flag: 97 responses corpus-wide; Turkish-Gemini-restricted flag: 48/60). Sensitivity analyses under alternative thresholds are reported in Supplementary Material S6.

Because generation-termination metadata (finish_reason for GPT and Claude, stop_reason for Claude, finishReason for Gemini) were not logged during the original data collection, the mechanistic cause of Turkish Gemini truncation—whether max-tokens limit reached, safety filter activation, or a Turkish-tokenisation inefficiency—cannot be determined from the corpus alone. Mechanistic hypotheses are therefore presented as hypothesis-generating rather than explanatory (see Discussion §Limitations).

As a sensitivity analysis, a secondary punctuation-based classifier was applied to all responses. This classifier was defined as: response ends without terminal punctuation (period, exclamation, question mark, closing quote, or closing bracket). Applied to the full corpus, this classifier flagged 163/360 responses. Cell-level flag rates were: Turkish Gemini 51/60 (85%); English Claude 52/60 (87%); English Gemini 16/60 (27%); Turkish Claude 23/60 (38%); English GPT-4o 19/60 (32%); Turkish GPT-4o 2/60 (3%). These cell-level rates reveal that the secondary classifier lacks specificity for incomplete generation: many responses in other cells end with Markdown formatting (bold closing tags, bullet-list endings) or paragraph structure rather than terminal punctuation. On Turkish Gemini specifically, the punctuation-based classifier (51/60, 85%) and the pre-specified word-count rule (48/60, 80%) agreed on 45/60 responses. This convergence supports the primary classification of Turkish Gemini responses as systematically truncated. In all other cells, stage-2 visual review confirmed that responses flagged by either classifier were brief but complete rather than truncated (see §Truncation classification).

Throughout this manuscript, we use the following terminology consistently: a response is called “truncated” when it was operationally identified as ending before natural completion (either by the pre-specified word-count rule, <250 words, or by the secondary punctuation/closing-convention classifier); “incomplete” is used interchangeably with “truncated” for the same operational construct; and “excluded” refers specifically to responses that were removed from the primary inferential analysis on the basis of that truncation identification. The pre-specified word-count rule was retained for the primary analysis because it was set a priori and does not rely on the punctuation heuristic; the secondary classifier is reported as a sensitivity analysis in Supplementary Table S12. The API metadata field indicating termination reason (finish_reason for OpenAI, stop_reason for Anthropic, finishReason for Google) was not logged by the data-collection script (ortho_collect_v2.py) at the time of the May 2026 collection, so a mechanistic classification by termination reason is not available from the current corpus; both classifiers we do report are operational (surface-level features of the returned text) rather than mechanistic (API-reported termination cause).

A sensitivity analysis using alternative word-count thresholds (150, 200, 250, 300 words) is reported in Supplementary Table S6; the pre-specified threshold of 250 words identified 48 responses, and reasonable variation around that threshold produced consistent identification of the systematic truncation pattern. Truncated responses were excluded from the primary analysis, leaving 312 responses (Claude EN: 60; Claude TR: 60; GPT-4o EN: 60; GPT-4o TR: 60; Gemini EN: 60; Gemini TR: 12). The truncation pattern is itself a substantive deployment finding and is reported separately in the Results.

### 2.7. Rater Calibration

A two-stage rater calibration procedure was planned before the main scoring phase and specified in the analysis protocol prior to any main-scoring data being collected. In stage one, the three raters completed a 15-response pilot. Analysis of the pilot showed systematic centrality differences between raters, with mean total scores of 3.31 (R1), 3.79 (R2), and 4.17 (R3) and an ICC(2,3) of 0.35 across the six dimensions. The largest gaps were in personalisation (range 1.71) and patient-centeredness (range 1.64). In stage two, a calibration meeting between the three raters clarified the personalisation and patient-centeredness rubrics with additional behavioural markers, after which all raters independently scored the full 312-response set.

The systematic centrality differences observed in the pilot were addressed with a pre-specified dimension-specific centring procedure applied uniformly across the main-scoring dataset. For each dimension, shift values were computed to align the two outer raters’ means with the median rater’s mean, and these shifts were applied to all scores from that rater on that dimension. Centred scores were clipped to the 1 to 5 range. Rank order of responses within each rater was preserved, so the procedure adjusted rater centrality without altering any rater’s relative judgement between responses. The uncalibrated (raw) and calibrated results are both reported in the Supplementary Material, and the direction and significance of every reported contrast is identical between the two analyses (Supplementary Table S1); calibrated results are presented in the main text for clarity.

### 2.8. Sample Size

A formal a priori power calculation was not performed because the design was fixed by clinical-coverage constraints rather than by anticipated effect sizes: the sample of 60 scenarios per model per language (360 total, 312 after truncation exclusion) was set at ten scenarios per concern category × model × language, which was chosen as the minimum practical count that balanced clinical coverage across the six concern categories against the scoring load for three independent raters on every response. Post hoc, the pre-specified English-only balanced comparison (three groups with *n* = 60 per cell) had 80% power to detect a medium between-model effect (f = 0.25) at α = 0.05; the effect sizes observed for the significant contrasts (η^2^ = 0.08 to 0.45) all sit well above this detectable-effect threshold. The two-way ANOVA on the balanced *n* = 300 subset had greater than 99% power for the observed language main effect (η^2^ = 0.34).

### 2.9. Statistical Analysis

Descriptive statistics (mean, SD, 95% CI) were computed for every model × language × dimension cell. Rater centring was pre-specified before any main-study outcome analysis and applied uniformly across all raters and all dimensions. The centring procedure was defined during the same 15-response pilot phase described in the Rater Calibration subsection above, after inspection of pilot rating distributions revealed rater-specific centrality differences (rater means differing by up to 0.6 points on a 5-point scale, in the absence of dimensional differences that could account for the shift). No main-study responses had been scored or inspected at the time the centring rule was defined; the pilot responses themselves were not included in the 312-response main-study analytic dataset. For the main-study data, each rater’s per-dimension mean across all 312 responses was subtracted from that rater’s raw score, and the grand mean across all raters and responses was added back, yielding a calibrated score that preserves within-rater variance and between-response contrasts while removing between-rater centrality shifts. The main analyses were run on both raw and calibrated scores and were concordant in every case (Supplementary S3 reports the uncalibrated results in full). This procedure was documented in the pre-analysis plan before the main-study data were unblinded to outcome analysis. Inter-rater reliability for the calibrated main-scoring data was assessed with ICC(2,3) (two-way random-effects, absolute agreement) using the Shrout and Fleiss formulation, and with Krippendorff’s α using ordinal weighting; 95% confidence intervals for ICC were obtained by non-parametric bootstrap (10,000 resamples).

Because the same 60 clinical scenarios were evaluated across models and languages, the primary between-model contrasts were fit as mixed-effects linear models with scenario as a random effect, accounting for the paired scenario structure across model and language conditions (full model tables in Supplementary S13; principal model contrasts summarised in the Results below). Mixed-effects fits used maximum-likelihood estimation (statsmodels 0.15.0, Powell optimiser), and length-adjusted specifications added response word count as a fixed-effect covariate. In parallel, one-way ANOVA and Tukey HSD post-hoc pairwise contrasts were computed on the calibrated mean score per response as a descriptive check; the ANOVA and mixed-effects results were concordant in direction and significance for every reported contrast. ANOVA assumptions were checked (Shapiro–Wilk for residual normality with Q–Q plot inspection; Levene’s test for homogeneity of variances); where either assumption was violated, results were confirmed with the non-parametric Kruskal–Wallis test with Dunn’s post-hoc, and the parametric and non-parametric estimates were concordant in every case.

Language effects were assessed using mixed-effects linear models with scenario as a random effect (dependent_variable ~ language + model + (1|scenario)), applied to the complete-factorial subset of Claude and GPT-4o responses (*n* = 240; Turkish Gemini responses were excluded from this analysis for reasons detailed under Truncation classification). Descriptive Welch t-tests are reported alongside for continuity with the earlier submission but are not the primary inferential test. Effect sizes were reported as η^2^ for ANOVA main effects and Cohen’s d (pooled standard deviation) for pairwise contrasts. To control the family-wise error rate across the six dimension-level ANOVAs, *p*-values were adjusted with the Holm–Bonferroni procedure; adjusted *p*-values are reported alongside the raw values in the Results.

Significance was set at α = 0.05 (two-sided). All analyses were run in Python 3.12 (numpy 1.26, scipy 1.13, pandas 2.2, pingouin 0.5.4, statsmodels 0.15.0).

To address the possibility that between-model differences in rubric scores were driven by between-model differences in response length rather than by architectural differences in communication quality, three additional analyses were performed. First, Pearson correlations between response length (word count) and each of the six calibrated dimension scores and the calibrated total score were computed on the full 312-response calibrated dataset. Second, on the balanced English-only sample (*n* = 180), one-way analysis of covariance (ANCOVA) was fitted with model as the categorical predictor and response length as a continuous covariate; adjusted marginal means at the grand-mean length were reported alongside the raw means. Third, on the same English-only sample, mixed-effects linear models were fitted with scenario ID as a random effect and model, model + length, and model + length + language as fixed effects, in nested comparisons; fitted with maximum likelihood (Powell optimiser) to enable likelihood-ratio tests between nested models. The length-adjusted coefficient for each between-model contrast and its statistical significance are reported in Supplementary S13. Ordinal mixed-effects sensitivity analyses using cumulative-link mixed models on the raw (uncalibrated) per-rater scores gave results concordant with the linear mixed models and are reported in Supplementary S13. Because the analytic sample for Turkish Gemini shrank to *n* = 12 after truncation exclusion, the design was no longer balanced across the model × language grid, and any pooled inference including Turkish Gemini would confound the model comparison with the truncation-driven sample-size imbalance. To keep the primary model comparison on a balanced footing, we designated the pre-specified primary analysis as the English-only three-model contrast (*n* = 60 per model, total *n* = 180), reported this contrast first in the Results, and treated the Turkish results as a secondary within-language contrast in which Turkish Gemini is reported descriptively rather than inferentially. Two secondary descriptive-check analyses were pre-specified: (i) a complete-factorial two-way ANOVA on the balanced Claude + GPT-4o × EN + TR subset (*n* = 240, four cells of *n* = 60 each, fully balanced), which supports language × model interaction inference; and (ii) an extended descriptive-check two-way ANOVA on the same subset plus Gemini English (*n* = 300, five cells of *n* = 60 each, missing one factorial cell), which places Gemini English descriptively alongside the balanced sample but does not constitute a balanced factorial design. Turkish Gemini (*n* = 12) is not included in either two-way analysis and is reported descriptively (mean and bootstrap 95% CI, see Supplementary Material S8). No unbalanced ANOVA that included the *n* = 12 Turkish Gemini cell was performed, because the resulting imbalance (60 versus 60 versus 60 versus 60 versus 60 versus 12) would have made the two-way ANOVA sensitive to the choice of sum-of-squares parameterisation. The extended descriptive two-way analysis (*n* = 300 for five cells) uses Type III sum-of-squares because the design is not balanced (Gemini has no Turkish cell); Type III SS ensures that model, language, and interaction terms are estimated adjusted for one another. Homogeneity of variance was verified with Levene’s test (all *p* > 0.05) and normality of residuals with Shapiro–Wilk (visually inspected via Q–Q plots). The primary inferential framework for both model-level and language-level comparisons remains the mixed-effects models described above; the two-way ANOVAs are reported for descriptive continuity with the earlier submission and for readers familiar with that framework. Data from the Turkish Gemini cell are reported descriptively (mean and bootstrap 95% CI) in Supplementary S8, and are not entered into any inferential test. Bootstrap 95% CIs (10,000 resamples) on Turkish Gemini means are provided in Supplementary Material S8 as a sensitivity check for readers who need a point estimate.

## 3. Results

### 3.1. Response Corpus and Truncation

**Figure 1:**
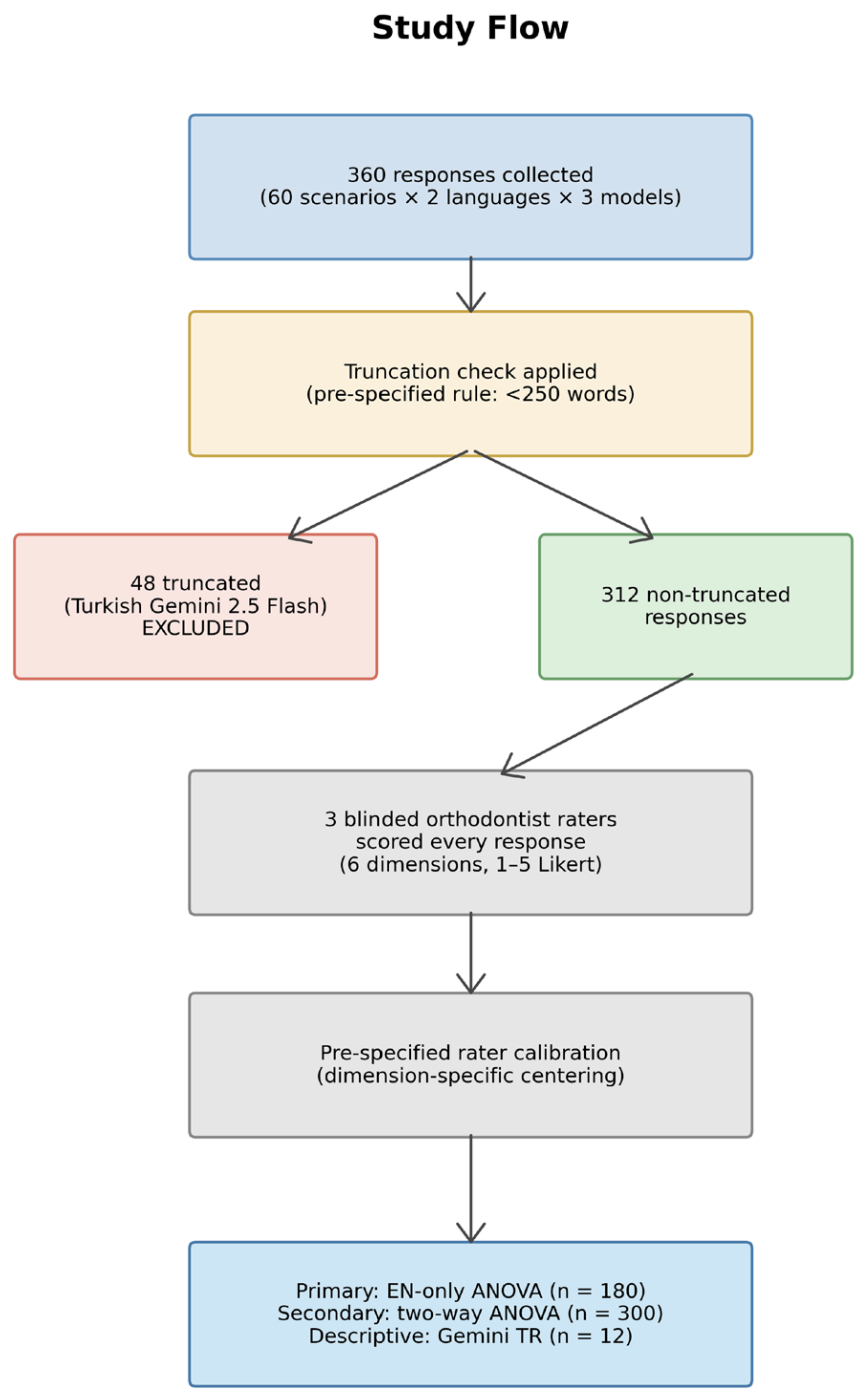
Study flow diagram: 360 responses collected, 48 truncated (Turkish Gemini 2.5 Flash) responses excluded, 312 scored by three blinded raters, calibration applied, main analysis conducted.

The corpus comprised 360 responses (60 scenarios × 2 languages × 3 models). Application of the truncation rule flagged 48 responses (13.3%). All were Turkish Gemini 2.5 Flash outputs, representing 80% (48/60) of that model–language cell. No truncation was observed in any other model–language combination. Mean word counts on the full 60-response Turkish samples were Gemini 147, Claude 250, and GPT-4o 190; the same model in English produced a mean of 547 words for Gemini EN with no truncation. Among the 12 non-truncated Turkish Gemini responses, the mean word count was 277, itself well below Gemini’s English mean of 547. The 312 non-truncated responses formed the primary analytic set (Table 1). The overall study flow, from the 360 collected responses to the 312 non-truncated responses in the analytic set, is illustrated in Figure 1.

### 3.2. Inter-Rater Reliability

Pilot ICC(2,3) was 0.35, with a between-rater centrality range of 0.88 total-score points. Applying the dimension-specific centring procedure lifted mean ICC(2,3) on the main dataset from 0.23 to 0.38. Reliability was heterogeneous across dimensions. Comprehensiveness (K) reached moderate to substantial agreement using Landis and Koch’s benchmarks (ICC(2,3) = 0.65), empathy (E) and patient-centeredness (HM) reached moderate agreement (ICC(2,3) 0.55–0.56), personalisation (P) reached fair to moderate agreement (0.45), and accuracy (D) and safety (G) had near-zero ICCs (0.03 and 0.01 respectively). Krippendorff’s α values were consistently below the 0.667 acceptable threshold, which is expected for a 5-point Likert scale in which the response-level variance is compressed relative to the rater-level variance; the ordering across dimensions is identical to that seen with the ICC. Krippendorff’s α followed the same pattern (Table 2). The near-zero accuracy and safety reliabilities reflect a restricted-range artefact rather than genuine rater disagreement. Across the pooled scores from all three raters, 88 per cent of accuracy scores and 92 per cent of safety scores were 4 or 5, and rater-level standard deviations spanned 0.18–0.78 for accuracy and 0.17–0.56 for safety across the three raters (median rater SDs 0.46 and 0.27, respectively). The intraclass correlation coefficient is defined as between-response variance divided by total variance; when between-response variance approaches zero, the coefficient approaches zero regardless of how closely raters agree in mean level. Because agreement on a compressed distribution is better summarised by allowing one-unit tolerance than by requiring identical scores, we report percent-agreement-within-one-point: for accuracy, this was 90 percent between R1 and R2, 98 percent between R1 and R3, and 97 percent between R2 and R3; for safety, the corresponding figures were 98, 99, and 99 percent. Both dimensions therefore showed high absolute rater concordance in effective terms despite a low ICC. This pattern also carries a substantive finding: the few responses were rated as clearly unsafe by this panel of three orthodontists (no consensus low-safety response was identified in the case-level analysis, see Supplementary Material S12.6), and the rating distribution was compressed toward the upper end of the scale on accuracy and safety, leaving the empathic, comprehensive, personalised, and patient-centered dimensions as the axes on which the models actually differed. The implications for future rubric design are addressed under Limitations.

### 3.3. Model-Level Differences

The primary between-model contrasts were fit as mixed-effects linear models with scenario as a random effect, using the balanced English sample (*n* = 60 per model, total *n* = 180) with the Turkish Gemini cell (*n* = 12) excluded from inferential analysis owing to truncation-induced imbalance and reported descriptively. One-way ANOVA and Tukey HSD post-hoc contrasts on the same balanced sample are reported alongside as a descriptive check; results were concordant in every case. On this balanced sample, Gemini 2.5 Flash produced the highest mean total score (4.04 ± 0.17), followed by GPT-4o (3.86 ± 0.11) and Claude Opus 4.5 (3.85 ± 0.11). One-way ANOVA on the English-only sample confirmed significant model effects on empathy (F(2,177) = 7.69, *p* < 0.001, Holm-adjusted *p* = 0.001, η^2^ = 0.08), comprehensiveness (F(2,177) = 71.56, *p* < 0.001, adjusted *p* < 0.001, η^2^ = 0.45), and personalisation (F(2,177) = 24.06, *p* < 0.001, adjusted *p* < 0.001, η^2^ = 0.21). On the English-only sample, accuracy (F(2,177) = 1.86, *p* = 0.16, adjusted *p* = 0.32, η^2^ = 0.02), safety (F(2,177) = 0.44, *p* = 0.64, adjusted *p* = 0.64, η^2^ = 0.005), and patient-centeredness (F(2,177) = 0.87, *p* = 0.42, adjusted *p* = 0.64, η^2^ = 0.01) showed no significant model differences, consistent with the near-ceiling scores those dimensions received.

A secondary descriptive analysis of the 312 non-truncated responses (Claude *n* = 120, GPT-4o *n* = 120, Gemini *n* = 72) is reported in Table 3. The mean total scores were Gemini 3.97 ± 0.22, Claude 3.70 ± 0.19, and GPT-4o 3.66 ± 0.23. For the twelve Turkish Gemini responses that completed successfully, the bootstrap 95 per cent confidence interval on the mean total score (10,000 resamples) was 3.57 to 3.70, and the corresponding CIs for empathy, comprehensiveness, and patient-centeredness were 3.07 to 3.38, 3.94 to 4.30, and 3.00 to 3.36, respectively (full CIs in Supplementary Material S8). Because this pooled sample confounds model comparison with the truncation-driven Turkish Gemini imbalance, its point estimates are reported for completeness only and are not used for inferential model contrasts. Shapiro–Wilk indicated mild deviations from normality in three of the six dimensions and Levene’s test flagged unequal variances in two; Kruskal–Wallis re-analysis was concordant with the parametric result for every significant contrast on the English-only sample (all Kruskal–Wallis *p* < 0.001), and the parametric estimates are reported for clarity.

A two-way ANOVA on the composite total score with model (3 levels) and language (2 levels) as factors was run on the balanced subset formed by the six model × language cells with *n* = 60 each; because Turkish Gemini contained only 12 non-truncated responses, this cell was excluded from the two-way analysis and reported descriptively (*n* = 300 for the two-way test). The two-way test found significant main effects of model (F(2,295) = 40.94, *p* < 0.001) and language (F(1,295) = 149.12, *p* < 0.001), and a significant but small model × language interaction (F(2,295) = 9.98, *p* = 0.002). The interaction reflected a slightly steeper English–Turkish drop for GPT-4o (0.40 total-score points) than for Claude (0.29 points) and did not alter the ranking of the three models within either language.

**Figure 2:**
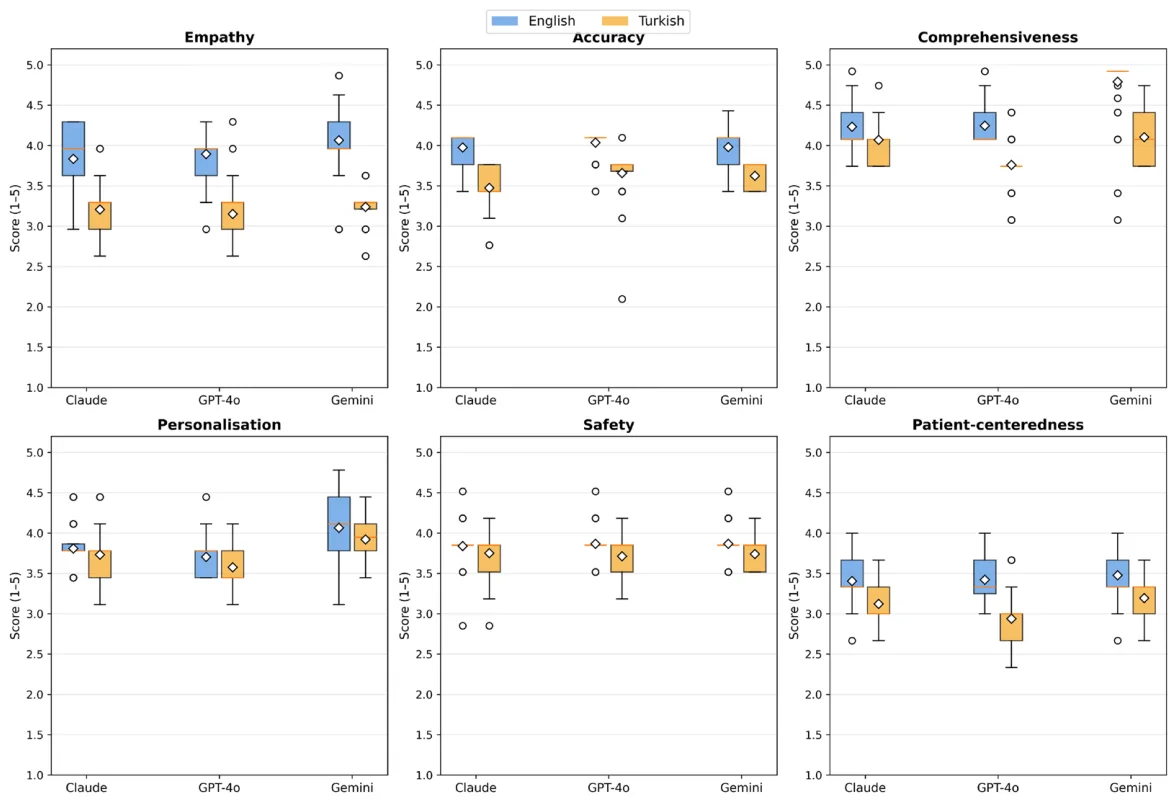
Box plots of dimension scores by model, stratified by language. Each panel shows one of the six scoring dimensions (E, D, K, P, G, HM) with English (blue) and Turkish (orange) responses for each model.

Tukey HSD post-hoc contrasts on the English-only sample clarified where the model differences lay. On empathy, Gemini scored significantly higher than both Claude (mean difference 0.23, adjusted *p* = 0.001, Cohen’s d = 0.65) and GPT-4o (mean difference 0.17, adjusted *p* = 0.016, d = 0.54), while Claude and GPT-4o did not differ (adjusted *p* = 0.58, d = −0.18). On comprehensiveness, Gemini exceeded Claude by 0.56 points (d = 1.87, adjusted *p* < 0.001) and GPT-4o by 0.55 points (d = 1.70, adjusted *p* < 0.001); Claude and GPT-4o did not differ. On personalisation, Gemini outscored Claude (d = 0.82, adjusted *p* < 0.001) and GPT-4o (d = 1.20, adjusted *p* < 0.001), and Claude scored slightly higher than GPT-4o (mean difference 0.11, adjusted *p* = 0.08, d = 0.40). For patient-centeredness, no post-hoc pairwise contrast reached significance after correction. The full dimension-by-dimension breakdown across model and language is presented in Table 3 and visualised in Figure 2.

### 3.4. Response Length as a Confounder

Response length varied substantially between models. On the English cell, mean word counts were 265 (Claude), 288 (GPT-4o), and 547 (Gemini)—Gemini responses were on average approximately twice the length of the other two models. Response length correlated strongly with rubric scores across every dimension: Pearson r = 0.84 for comprehensiveness, 0.75 for total score, 0.56 for empathy and personalisation, and 0.43 for patient-centeredness (all *p* < 0.001), and 0.26 and 0.21 for accuracy and safety (both *p* < 0.001). Length alone explained approximately 56% of the variance in total scores.

Because Gemini differed from the other two models principally in output length, we tested whether the Gemini advantage on the English sample persisted after length adjustment. A one-way ANCOVA on the English sample with response length as a covariate reduced the model effect on total score from F(2, 177) = 38.66 (*p* < 0.001, unadjusted) to F(2, 176) = 2.61 (*p* = 0.076, length-adjusted); the length covariate itself was highly significant (F(1, 176) = 114.60, *p* < 0.001). Length-adjusted marginal means for total score were 3.94 (Claude), 3.93 (GPT-4o), and 3.87 (Gemini)—that is, after length adjustment, Gemini scored numerically lower than the other two models rather than higher. The homogeneity-of-regression-slopes assumption underlying ANCOVA was tested for each dimension by adding a model × response-length interaction term. The assumption was supported for total score (F(2, 174) = 2.20, *p* = 0.114), empathy (*p* = 0.50), accuracy (*p* = 0.76), personalisation (*p* = 0.39), safety (*p* = 0.86), and patient-centeredness (*p* = 0.69). For comprehensiveness, the interaction was significant (F(2, 174) = 10.58, *p* < 0.001), indicating that the score–length slope differs by model on this dimension; the length-adjusted marginal comparison of comprehensiveness should therefore be interpreted with caution and is best considered together with the per-model within-length regression estimates reported in Supplementary Table S13.

A mixed-effects model on the English sample (total ~ model + length + (1|scenario)) yielded the same pattern (Table 4). Without length adjustment, Gemini exceeded Claude by β = +0.192 total-score points (*p* < 0.001); with length in the model, the coefficient reversed to β = −0.065 (*p* = 0.017). At the dimension level, the Gemini-vs-Claude advantage that was significant without length adjustment on empathy (β = +0.232, *p* < 0.001), comprehensiveness (β = +0.559, *p* < 0.001), and personalisation (β = +0.256, *p* < 0.001) was no longer significant with length in the model (β = −0.06 to −0.10, all *p* > 0.10). On patient-centeredness, the coefficient reversed from a non-significant β = +0.072 to a significant β = −0.153 (*p* = 0.021), indicating that after length adjustment, Gemini scored lower than Claude on patient-centeredness. Accuracy and safety showed no model effect either before or after length adjustment. The GPT-4o-vs-Claude comparison was non-significant both with and without length adjustment on every dimension. Full mixed-effects tables are provided in Supplementary S13.

The interpretation of these results is examined in the Discussion; briefly, they indicate that the raw between-model differences on the English sample are largely explained by Gemini producing longer outputs rather than by architectural differences in communication quality, and that on a length-normalised basis Gemini is either equivalent to or lower than Claude and GPT-4o.

### 3.5. Language Effect

English prompts scored substantially higher than Turkish prompts across all six dimensions. The primary inferential analysis was a mixed-effects linear model with scenario as a random effect, applied to the complete-factorial subset (Claude + GPT-4o × EN + TR, *n* = 240). This showed a large and significant English–Turkish gap on total score (β_TR = −0.343, SE = 0.014, *p* < 0.001), with a language × model interaction (β = −0.106, SE = 0.026, *p* < 0.001) indicating that the Turkish disadvantage was more pronounced for GPT-4o than for Claude. Per-dimension mixed-effects estimates of the Turkish-versus-English contrast were largest for empathy (β = −0.686, SE = 0.035, *p* < 0.001), moderate for accuracy (β = −0.439, SE = 0.026, *p* < 0.001), patient-centeredness (β = −0.383, SE = 0.033, *p* < 0.001), and comprehensiveness (β = −0.324, SE = 0.030, *p* < 0.001), and smaller for safety (β = −0.122, SE = 0.026, *p* < 0.001) and personalisation (β = −0.103, SE = 0.026, *p* < 0.001). For descriptive continuity with the earlier submission, Welch t-tests on the marginal means (English *n* = 180, Turkish excluding Gemini *n* = 120) yielded total-score means of 3.92 versus 3.51 (Welch’s *t*(284) = 23.89, *p* < 0.001, Cohen’s d = 2.71); the corresponding dimension-level Cohen’s d values were empathy 2.22, accuracy 1.87, comprehensiveness 1.38, patient-centeredness 1.26, personalisation 0.60, and safety 0.61. Because the English and Turkish prompts represent translations of the same clinical scenarios, the Welch analysis does not account for the paired scenario structure and is provided for descriptive comparison only; the mixed-effects estimates are the primary inferential result.

**Figure 3:**
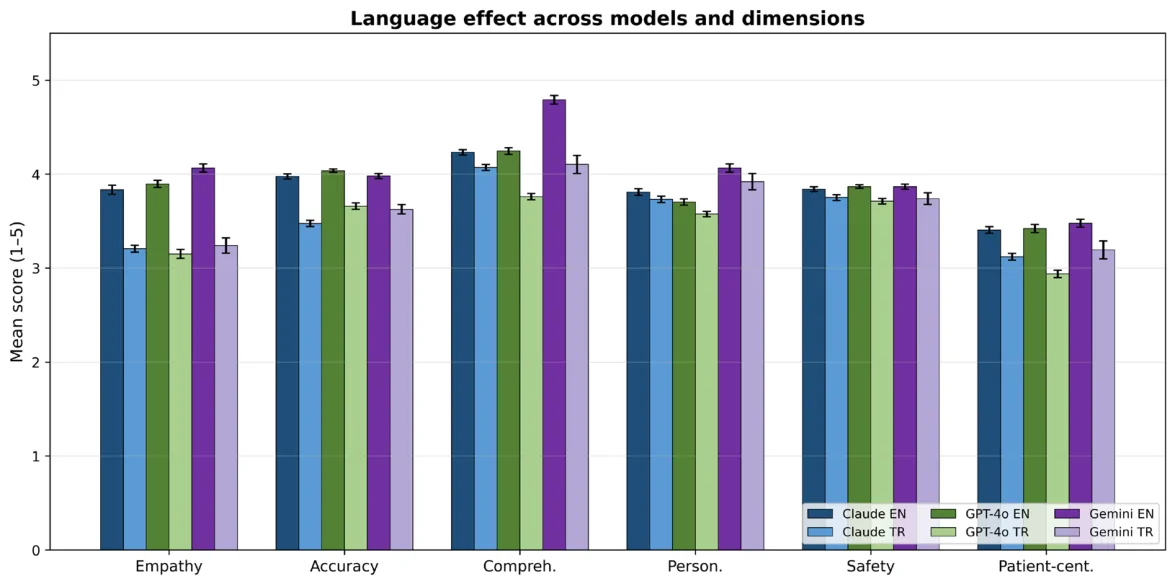
Language effect across models and dimensions. Bar heights show mean scores; error bars show standard errors. The English–Turkish gap is visible in every dimension of every model.

The language gap was present within every model (Figure 3), which makes it unlikely to be an artefact of model–language composition. Empathy dropped by 0.63 points from EN to TR for Claude (d = 1.90), by 0.74 for GPT-4o (d = 2.27), and by 0.83 for Gemini (d = 2.49, based on the reduced *n* = 12 Turkish sample). All within-model drops were significant at *p* < 0.001. Accuracy dropped by 0.50 for Claude, 0.38 for GPT-4o, and 0.36 for Gemini. Patient-centeredness dropped by 0.28 for Claude, 0.48 for GPT-4o, and 0.28 for Gemini. GPT-4o TR patient-centeredness of 2.94 was the single lowest cell in the entire matrix.

**Figure 4:**
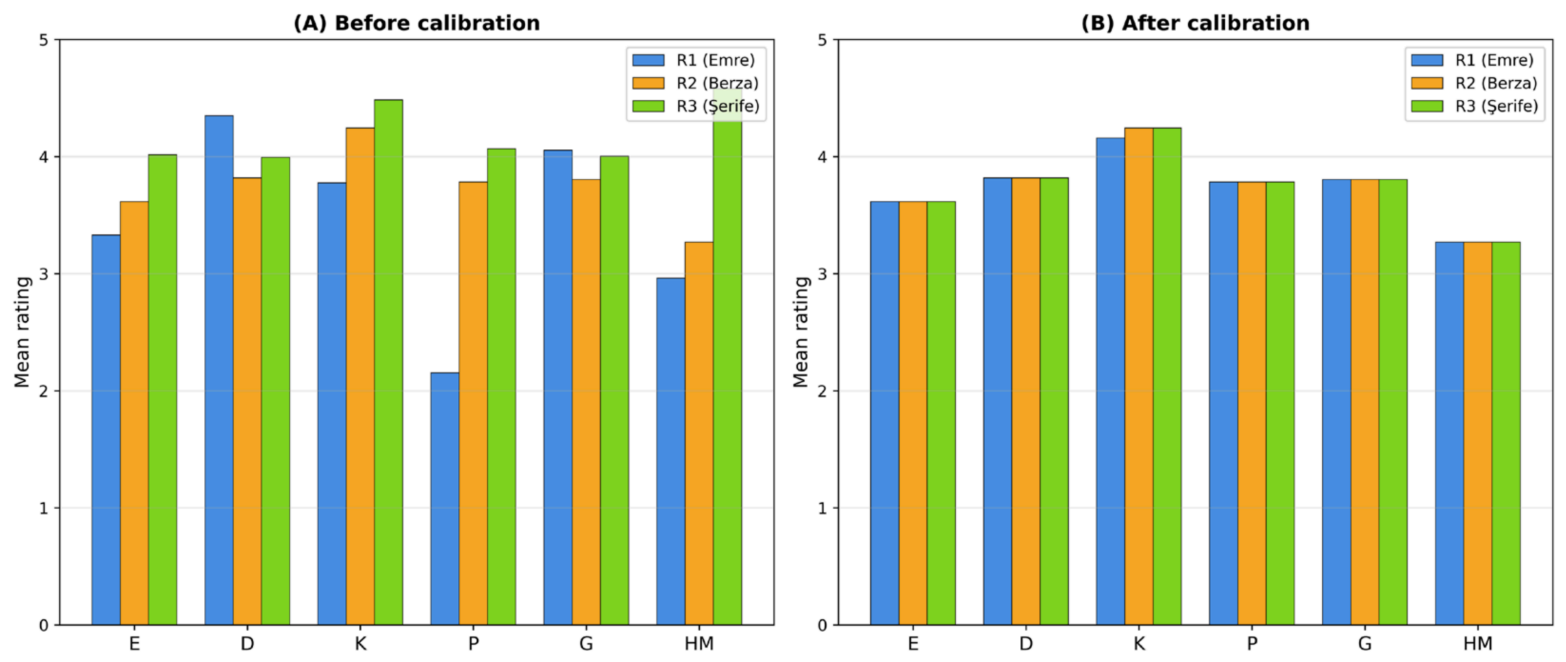
Rater bias visualisation before (**A**) and after (**B**) the dimension-specific centring procedure. Bars show per-rater means by dimension.

The effect of the dimension-specific centring procedure on per-rater means is illustrated in Figure 4 (panel A: before centring; panel B: after centring).

### 3.6. Model × Language Interaction

On the English-only sample, Gemini > Claude ≈ GPT-4o (4.04 vs. 3.85 vs. 3.86). The formal two-way ANOVA on the balanced Claude + GPT-4o × EN + TR subset (Turkish Gemini excluded because of the truncation-driven imbalance) confirmed both main effects (model *p* < 0.001, language *p* < 0.001) and a small but significant model × language interaction (*p* = 0.002), reflecting a slightly steeper English–Turkish drop for GPT-4o (0.40 points) than for Claude (0.29 points). For descriptive completeness, the twelve non-truncated Turkish Gemini responses had a mean total score of 3.64, which sits within the range spanned by Claude TR (3.56) and GPT-4o TR (3.47), but the twelve-response sample is too small to place a stable model ranking on it, and no inferential claim is made about Gemini in Turkish.

### 3.7. Dimension-Level Pattern Across the Corpus

Averaged across all model–language cells, comprehensiveness was the highest-scoring dimension (K = 4.22), followed by accuracy (D = 3.82), safety (G = 3.80), personalisation (*p* = 3.78), and empathy (E = 3.62). Patient-centeredness was the lowest-scoring dimension across the entire corpus (HM = 3.27), a full 0.95 points below comprehensiveness. This pattern held for every model and every language.

Category-level breakdowns are summarised in Table 5 and reported in full in Supplementary Table S7. Patient-centeredness was the lowest-scoring dimension in every one of the six concern categories (means 3.16 to 3.53), replicating the corpus-level finding at the category level. Aesthetics and social concerns produced the lowest mean total score across models (3.70), and cost and decision-making scenarios the highest (3.81); the between-category range on total score was 0.11, small relative to the between-model range on the English-only sample (0.19) and the between-language range (0.41). Between-category variation was therefore secondary to the between-model and between-language effects described above, and because category-level cells contained only 10 responses per model per language, category comparisons are reported descriptively rather than as inferential contrasts.

### 3.8. Truncation as a Deployment-Relevant Failure

The systematic truncation of Turkish Gemini 2.5 Flash outputs is a separate finding whose weight is independent of the scoring analysis. Eighty per cent of that model–language cell terminated mid-sentence, mid-word, or well short of the corpus’s typical response length. Gemini’s mean Turkish word count of 147 across the full 60-response sample sits against its own mean English word count of 547, a nearly fourfold gap that is not observed for either Claude (250 TR vs. 265 EN) or GPT-4o (190 TR vs. 288 EN). This is a model-specific and language-specific output-limit failure that would materially affect any Turkish patient-facing deployment of Gemini 2.5 Flash and is not visible in aggregate performance metrics.

## 4. Discussion

### 4.1. Principal Findings

Three findings stand out. On the responses that completed successfully, Gemini 2.5 Flash produced the highest empathic scores in English. The same advantage did not carry over to Turkish: 80% of Turkish Gemini responses terminated mid-generation and were dropped from the primary comparison, and the twelve that remained were too few to run an inferential contrast. So a statement like “Gemini is the most empathic model” applies specifically to its completed English outputs, and does not extend to Turkish in the current configuration.

The English–Turkish gap in the two languages tested was large and consistent. All three models scored substantially lower on Turkish than on English on every dimension, with effect sizes reaching Cohen’s d = 2.22 for empathy. Since the models are the same on both sides of the comparison, the gap does not reduce to model choice.

Patient-centeredness was the weakest dimension across the entire corpus, sitting almost a full point below comprehensiveness. That is: current LLMs answer orthodontic questions competently, but they score lower on this study’s operational measure of patient-centred communication—which captures decision-sharing language, elicitation of patient preferences, and framing of options—and this is where the models have the most room for improvement.

### 4.2. Response Completion and Response Quality Are Two Separate Outcomes

Because the missingness in this study is informative (i.e., not random)—48 of the 48 excluded responses came from a single model–language cell. Two outcomes have to be kept apart. One is whether a response was generated to completion; the other is how well a completed response scored. These are separate questions, and we report them separately. On completion, one cell dominates: 80% of Turkish Gemini outputs failed to complete on stage-2 visual review; the corresponding stage-1 secondary flag rate (absence of terminal punctuation) was 85% for Turkish Gemini and 3–87% across the other five cells. Because the secondary classifier detects punctuation-based closure rather than semantic completeness, it is high-sensitivity but low-specificity for incomplete generation—many complete responses in other cells end with Markdown formatting or paragraph structure rather than terminal punctuation—and it should not be interpreted independently of stage-2 review. On quality, the analysis rests on the 312 completed responses only; wherever we say “model X scored highest,” we mean the quality of what was completed, not of what was attempted. Terms are used consistently: “completion,” “truncation,” and “excluded responses” refer to the first outcome, and score-level statements to the second.

### 4.3. Why Length Drives the Raw Between-Model Differences, and Why Gemini Still Fails in Turkish

Gemini’s raw advantage on comprehensiveness, empathy, and personalisation on the English sample tracked its much larger English response length (mean 547 words vs. 265 for Claude and 288 for GPT-4o). Longer responses covered more of the question, provided more surrounding context, offered more concrete examples, and gave more space for empathic framing—all of which the raters, working with a rubric that rewards thoroughness and warmth, would have valued. When response length was entered as a covariate in the mixed-effects model, the Gemini advantage on empathy, comprehensiveness, and personalisation was eliminated (all *p* > 0.10), the total-score coefficient reversed sign, and Gemini’s patient-centeredness coefficient became significantly negative. This pattern is diagnostic: raw rubric scores were dominated by output length, and Gemini’s unadjusted advantage on the English sample is strongly associated with, and statistically attenuated after adjustment for, response length in patient communication.

The same generation behaviour was not observed in Turkish. All three models were queried through a stateless single-turn API with an identical maximum output-token budget (2000 tokens); nonetheless, Gemini’s mean Turkish word count across all 60 responses was 147, versus 250 for Claude and 190 for GPT-4o, and 80% of Gemini’s Turkish outputs terminated mid-generation without sentence-final punctuation. Because the token limit was identical across models, the disproportionate Gemini truncation is not a straightforward “hit the limit” story; two mechanisms are consistent with the observed pattern. One mechanism is tokeniser efficiency: Turkish is an agglutinative language that typically fits fewer surface words into a given tokeniser budget than English does, and per-model tokeniser efficiency for lower-resource and morphologically rich languages is known to differ substantially between LLM providers [20]. If Gemini’s tokeniser is markedly less efficient for Turkish than for English, the same 2000-token budget would allow proportionally fewer Turkish words for Gemini than for Claude or GPT-4o. The other is a possible model-side generation policy: Gemini’s handling of Turkish clinical text may terminate earlier than for English clinical text for reasons independent of the token budget (safety filtering, agglutinative-language handling). Distinguishing between these hypothesised mechanisms would require a controlled study varying the token budget and instrumenting each provider’s finish-reason metadata, which we did not have access to in this study; both mechanisms translate to the same clinical takeaway.

The clinical takeaway is that Gemini 2.5 Flash, in its May 2026 API configuration, cannot be recommended for patient-facing Turkish deployment without per-language completeness monitoring. The English-cell Gemini advantage that a rubric-based evaluation would report does not survive length adjustment and does not carry over to the Turkish deployment condition; a system that ranks highest on an English rubric can be the same system that most systematically fails to complete a Turkish output in a more open task. This underscores that FAQ-style single-language evaluations, however well-executed, cannot substitute for open-response bilingual evaluation when the question is whether a model is safe to deploy for patient-facing communication.

### 4.4. The English–Turkish Gap Is Consistent Across Every Model, Not Model-Specific

The most consequential finding is the language gap. Every one of the three models scored substantially lower on Turkish prompts than on English prompts across every scored dimension. The gap was largest for empathy (Cohen’s d = 2.22 in the pooled sample, and d = 1.90 for Claude, 2.27 for GPT-4o, and 2.49 for Gemini within each model) and smallest for safety (d = 0.60), but was significant everywhere. This is not a model choice problem, since all three models produced the gap. A plausible contributing factor is that English orthodontic and general medical text likely dominates the training corpora of all three models, though we did not measure the composition of any model’s training data and this observation should be regarded as a hypothesis rather than as a demonstrated cause. Whatever the source, the resulting response quality gap on Turkish prompts is a systematic bias that any clinician-facing deployment must reckon with. Similar cross-lingual quality gaps have been reported for other LLMs on general healthcare queries [15], for medical licensing exam questions [21], and for consumer-health advice [22]. Our data extend this pattern into orthodontic empathic communication and quantify it at Cohen’s d = 2.22 for empathy in a domain where empathic response quality is not incidental. From a policy standpoint, aggregate LLM performance metrics published in English obscure a very large gap that appears the moment the same system is asked to serve non-English patients.

Two important limitations of this language finding must be kept in view. A first boundary is language coverage: only English and Turkish were evaluated in this study, so a broad multilingual quality gap cannot be inferred from a two-language design, and the finding should be read narrowly as an English–Turkish contrast under the evaluated conditions. Extension to a broader panel of languages—including languages that are structurally close to English, structurally distant from English, and lower-resource in typical LLM training corpora—would be needed to test whether the English–Turkish pattern generalises. A second boundary concerns causal decomposition: the observed English–Turkish difference could arise from several distinct sources—differential model performance in the two languages, linguistic properties of Turkish (agglutination, morphology, script), pragmatic or lexical equivalence of the translated scenarios, effects of the translated prompt on model behaviour, and rater expectations for Turkish clinical communication—and the present design cannot distinguish among these contributing factors. The conclusion that English-only evaluation cannot automatically be assumed to generalise to Turkish is well supported by the present data; broader claims about multilingual LLM performance should be treated as hypotheses requiring confirmation in additional languages and clinical contexts.

### 4.5. Patient-Centeredness Is the Weakest Dimension

Patient-centeredness scored 3.27 across the corpus, a full 0.95 points below comprehensiveness (4.22). The pattern was consistent across all three models, both languages, and all six concern categories: patient-centeredness was the lowest-scoring dimension in every category-level cell (Table 5). Read against the behavioural markers used in scoring (shared-decision-making phrases; absence of paternalistic constructions), this pattern is instructive. Current LLMs answer orthodontic questions competently and cover the relevant clinical ground. But they default to a paternalistic frame that treats the patient as a recipient of information rather than a partner in a decision. The dimension that is arguably most central to modern patient-centred care [18,19] is the one on which today’s frontier models perform worst. This is a training and system-prompt problem, and it is likely tractable: patient-centeredness is easier to lift with prompt engineering than accuracy is. But no user-facing deployment we are aware of currently does this.

### 4.6. Comparison with Prior Work

Prior LLM evaluations in orthodontics have measured factual accuracy against clinical guidelines [11,12], readability of outputs [13], single-turn question answering [14], pretreatment patient-education content quality [23], and orthodontic knowledge assessment across multiple LLMs [24], alongside AI systems supporting treatment monitoring and retention [25]. The most influential study in the neighbourhood, Ayers et al. on Reddit r/AskDocs [2], found ChatGPT responses rated as more empathic than physician responses in 79% of pairwise comparisons—but on English prompts, general medical questions, a single model version, and with no cross-language check. Our study extends the empathy line of work into orthodontics, into a three-model comparison, and, most importantly, into a bilingual design that shows what an English-only evaluation cannot.

Studies of Gemini on English orthodontic knowledge tasks have reported acceptable accuracy [12,14], and multi-LLM benchmarks on English orthodontic knowledge items show broadly comparable factual performance across leading systems [24]; the same model in the same task family fails almost completely on Turkish free-form patient communication in our data. Those two observations can only be reconciled by evaluating each language separately.

The closest prior study to ours is Yüceer-Çetiner et al. [26], who evaluated ChatGPT-4, Gemini 2.5 Pro, and Claude Sonnet 4 on twenty patient-oriented FAQ items on orthognathic surgery in Turkish. Two of their findings converge with ours; a third marks the boundary. They reported Gemini outperforming ChatGPT and Claude on Global Quality Score, empathy, and clinical appropriateness on their Turkish FAQ set (all p < 0.001). Our English sample shows a similar unadjusted direction, with Gemini highest on empathy and comprehensiveness before length adjustment—although in our data the direction is limited to English, since Turkish Gemini could be assessed only descriptively (*n* = 12 after truncation exclusion) and did not clearly out-score Claude or GPT-4o in that small sample. The unadjusted Gemini-highest direction therefore holds across two different task types (open-response scenarios and curated FAQs), and two Gemini configurations (2.5 Flash in ours, 2.5 Pro in theirs); whether it survives length adjustment in the Yüceer-Çetiner corpus is an open question their study did not address. Their Gemini responses were rated clinically appropriate at high rates; our accuracy and safety scores likewise sit near ceiling for all three models. The boundary: their FAQ items are short and single-turn, and would not have surfaced the Turkish output-truncation failure we see in Gemini 2.5 Flash on a more open task, because FAQ answers rarely exceed the output budget. The two studies are compatible: Gemini can be rated highest on its Turkish outputs when those outputs complete, and it can be the model that most systematically fails to complete a Turkish output in a longer task. FAQ evaluations, however well-executed, cannot substitute for open-response evaluation when the deployment question is whether a model is safe for patient-facing use.

### 4.7. Clinical and Policy Implications

The three findings above point to a single deployment principle. No LLM should be released for patient-facing clinical use on the basis of English performance alone. Per-language quality assurance is a prerequisite, not an optional extra, and needs to cover both output completeness (were the responses actually finished?) and output quality (were they warm, personalised, and respectful?). The Turkish Gemini truncation finding is unambiguous: aggregate metrics that pool languages hide substantial single-language failures. The English–Turkish empathy gap observed here is subtler but at least as important: models can pass an English safety and accuracy audit and still deliver a systematically colder, less personal, and more paternalistic experience to non-English-speaking patients. Clinicians and clinics considering LLM deployment for patient triage, out-of-hours reassurance, or education should demand per-language evaluations from vendors and should not accept English performance as a proxy.

### 4.8. Strengths and Limitations

The main strengths of the study are the three blinded independent raters drawn from three different institutions, the bilingual design, systematic scenario coverage across the six main orthodontic concern categories, an a priori rubric with behavioural markers, a formal calibration procedure, and open data and analysis code.

The study has several limitations. The near-zero ICC on the safety dimension deserves a specific case-level examination beyond the mean-score comparison. Aggregating the three raters’ safety scores per response (Supplementary S12.4), no response received a mean safety score of 2 or lower, only two responses had a mean between 2 and 3, and no response did all three raters independently assign a low safety score (≤3). The three individual low-safety ratings that appear in the raw score distribution were isolated single-rater flags on responses that the other two raters scored as 4. That is: at the resolution the three-rater panel and this rubric can support, there is no evidence of concentrated safety failures. As reported in Supplementary S12.6, three individual ‘1’ safety ratings appear in the corpus, and all three came from the same rater (R2); the other two raters scored those same responses 4 or 5. The three responses fell in two categories (Pain & Comfort, Invasive Procedures) and two models (Claude Opus 4.5, GPT-4o); none reached consensus low safety, and no response had a mean composite safety below 2. This reads as isolated rater dissent, not a shared safety concern on the standardised orthodontic scenarios evaluated here. The safety-dimension conclusion is therefore that current LLM outputs on these scenarios do not contain consensus safety failures, not that any observed safety difference between models is statistically supported—a distinction that follows directly from the near-zero safety ICC discussed in the reliability paragraph above. A larger rater panel or a targeted analysis of clinically meaningful error types (incorrect clinical recommendations, inappropriate reassurance, failure to recommend professional assessment, unsafe self-management advice) on a wider scenario set would be needed to test safety differences between models with adequate power; we frame this as future work rather than as a limitation of the mean-safety-score comparison reported here.

The scenario set and the prompting strategy have limits on ecological validity that are worth naming explicitly. The 60 scenarios were authored by clinicians to be representative of orthodontic patient concerns across six categories and were held constant across the three models and both languages so that between-model and between-language differences could be attributed to the models rather than to variation in the input. Real-world patient prompts, by contrast, vary substantially in wording, health literacy level, emotional expression, completeness of information, and conversational history; some are terse, some are rambling, some contain misinformation the patient has read elsewhere, and some are part of a longer exchange rather than a first turn. The standardised, single-turn, clinician-authored scenarios used here favour experimental control at the cost of that real-world variability, and the between-model rankings we report should be understood as rankings on the standardised task rather than as predictions of how the same models would rank on real patient traffic. Similarly, the “no disclaimer” prompt instruction, justified above for deployment realism and between-model comparability, standardises out one form of variability (the extent to which each model would spontaneously add its own disclaimer style) at the cost of removing a behaviour that could be relevant in a real deployment where disclaimers are handled at the model rather than the platform layer. One further concern about the no-disclaimer instruction is that it may itself have shaped how much models wrote: if a model’s default disclaimer content moved into the body of the reply instead of being suppressed, that would show up as extra length. Our length-adjusted analyses (Table 4) address this: the vanishing of between-model differences once length is controlled is consistent with that mechanism, though it does not prove it. Extension of this evaluation framework to a corpus of de-identified real patient prompts, and to conditions in which disclaimers are permitted, are important complementary studies.

The scenarios are hypothetical rather than live patient interactions; findings may not fully generalise to real clinical use, though the standardised design permits controlled comparison. The total score reported alongside dimension-level scores is a simple unweighted arithmetic mean of the six calibrated dimension scores; it is not a validated unidimensional scale, and the six dimensions are conceptually distinct rather than interchangeable. The summed composite is internally consistent for this corpus (Cronbach’s α = 0.75; first principal component on the six dimension-level scores accounts for 54.8% of the variance, remaining components each under 15%; full correlation matrix in Supplementary S12.5). Even so, a model could theoretically compensate for a lower safety score with a higher empathy or comprehensiveness score and reach a similar total, even though these dimensions are not clinically equivalent—a lower safety score is not a defect that a higher empathy score could offset. For that reason, the dimension-level results are the primary outcomes in this study, and the total score is retained as a descriptive summary that makes the overall pattern across the six dimensions easier to communicate rather than as a validated global measure of communication quality. All between-model and between-language conclusions in the Results and Discussion are reported at the dimension level as well as at the total-score level, and no clinical inference is drawn from the total score that is not supported by the underlying dimension-level pattern.

The rater panel was drawn from a single country. This was a design choice, not a convenience: Turkish was one of the evaluated languages, and scoring Turkish empathic tone and patient-centeredness reliably needs native fluency in Turkish. Using non-Turkish raters for the English cells would have swapped a shared-panel confound for a rater-by-language confound, which is worse. English proficiency of the panel was checked in the pilot, and no scoring disagreement traced back to English comprehension, but we did not administer a formal English test such as TOEFL or IELTS; that is a real gap. Empathy and patient-centeredness are also culturally shaped, so absolute score levels should not be exported to other clinical cultures without checking; the direction of the model and language effects, though, does not turn on this and should replicate.

Only one response per prompt was collected, so run-to-run variability was not quantified. This is a substantive limitation. At the default temperature of 1.0, the same prompt can produce responses that differ in wording, in the order of points, and in the specific examples used, and how much this shifts rubric-level scores has not been settled in the literature. The principal findings—Gemini highest on empathic quality in English, systematic Turkish truncation for Gemini, the English–Turkish gap across all three models, weak patient-centeredness across all three—are large enough that a single-run design can detect them, but we cannot put a variance band on them. The 80% Turkish Gemini truncation rate under a single run is a strong enough signal that stochastic variation across runs would not move it to zero; a multi-run replication would narrow the true rate around that figure and would show whether some scenarios truncate more reliably than others. The same caveat applies to the between-model word counts: the gaps we report (Gemini English 547 vs. Turkish 147; Claude English 265 vs. Turkish 250) are too large to close with sampling noise alone, but the exact numbers are point estimates. Sampling five to ten runs per prompt, reporting mean and range at the response level, and then aggregating as we have would be a stricter test and is the first-priority extension of this work.

The analytic sample for Turkish Gemini shrank to *n* = 12 after the truncation exclusion, which limits power for that specific cell; all Turkish Gemini contrasts are therefore reported descriptively.

The rater centring procedure adds a modelling step. It was pre-specified before the main analysis, it preserved the rank order of responses within each rater, and every reported contrast has the same direction and significance whether calibrated or uncalibrated scores are used (Supplementary Table S1); both versions are reported for transparency.

This section distinguishes explicitly between three concepts often collapsed in discussions of inter-rater reliability: agreement (whether rater scores land close together), reliability (whether rater scores discriminate between responses), and restricted range (whether the underlying variance is compressed enough for either to be measurable). Absolute agreement was moderate at best (ICC(2,3) 0.55–0.65 for empathy, comprehensiveness, and patient-centeredness; 0.45 for personalisation), and Krippendorff’s α values sat below the 0.667 tentative threshold. ICC and Krippendorff measure different things: ICC(2,3) is the reliability of the mean of three raters, which is what enters our analyses, while Krippendorff’s α is defined on individual-rater judgements and is known to run conservative on short ordinal scales with compressed variance. Because our inferential contrasts use the per-response mean of three raters, ICC is the more relevant index here. Several lines of evidence indicate the reported effects are not artefacts of low agreement. Direction and significance of every contrast are identical between calibrated and uncalibrated scores (Supplementary Table S1), which would not hold if rater disagreement were driving results. When each rater is analysed separately, the ranking of the three models on empathy, comprehensiveness, and personalisation is the same for every rater on the English-only sample. The effect sizes we report (η^2^ of 0.08–0.45 for the significant English-only model contrasts, d = 2.71 for the language contrast on the total score) are also too large to be explained by rater noise. The near-zero ICCs for accuracy (0.03) and safety (0.01) reflect restricted range rather than genuine disagreement—88% of accuracy scores and 92% of safety scores were 4 or 5, and rater agreement within one Likert point was 90–98% for accuracy and 98–99% for safety. Model comparisons on accuracy and safety were consequently non-significant, which fits with three raters agreeing that responses across the corpus were near-uniformly accurate and safe. Future rubrics will either need wider anchor definitions for accuracy and safety, or drop them from frontier-LLM comparisons on this class of task.

LLM performance is a moving target. What we measured is the behaviour of three specific model versions—Claude Opus 4.5, GPT-4o, and Gemini 2.5 Flash, all as of the May 2026 API snapshot—and none of these will remain in production forever. The specific magnitudes should be read as a snapshot. The more durable contributions of the study are the methodology (bilingual rubric-based comparison with pre-specified analysis), the demonstration that English-only evaluation misses a very large Turkish gap in this domain, and the identification of patient-centeredness as the persistent weak point. Whether the exact numbers hold for GPT-5, Gemini 3.0, or Claude 5 is a question for replication; the code and rubric in Supplementary Material S2, S4, and S5 are structured to allow direct re-run against new model versions.

### 4.9. Future Work

Priority next steps are, first, a multi-run replication design that samples five to ten responses per prompt to quantify LLM run-to-run variability at the response level (the first-priority extension noted in Limitations); prospective evaluations with real patients rather than hypothetical scenarios; expansion of the bilingual design to additional languages; longitudinal LLM re-evaluation as new model versions are released; and adversarial testing focused specifically on patient-centeredness given its low performance across all models. On the policy side, per-language quality-assurance frameworks for patient-facing LLM deployment in clinical settings warrant development, and per-language completeness monitoring—at minimum—is a reasonable near-term recommendation. We frame this as a recommendation for future evaluation frameworks rather than as a policy prescription directly established by the present data, which are cross-sectional, cover two languages and three API-snapshot model versions, and use a single generation per prompt; broader validation across more languages, more model versions, real (rather than standardised) patient prompts, and multi-run designs would be needed before regulatory or mandatory deployment recommendations could be made.

## 5. Conclusions

We scored 312 blinded responses from three current LLMs to standardised orthodontic patient scenarios in English and Turkish. Three points stand out.

Rubric-based scoring of open-response LLM outputs is strongly associated with response length. Gemini’s raw advantage on the English sample was eliminated after length adjustment; on a length-normalised basis, the three models were indistinguishable on empathy, comprehensiveness, and personalisation, and Gemini scored lower than Claude on patient-centeredness. Unadjusted rubric comparisons of frontier LLMs on this class of task may therefore mainly reflect output length differences rather than architectural differences, and future evaluations should routinely report length-adjusted results.

The English–Turkish quality gap in the two languages tested was large, consistent across every model and every dimension, and robust to length adjustment. Gemini 2.5 Flash additionally truncated 80% of its Turkish outputs in the May 2026 API configuration under a token budget that was adequate for the other two models. Per-language quality assurance—for both output completeness and length-normalised content quality—is a prerequisite for patient-facing LLM deployment; English-only performance is not a safe proxy.

Patient-centeredness was the weakest dimension across the entire corpus in both unadjusted and length-adjusted analyses. The models answer orthodontic questions competently, but they score lower on this study’s operational measure of patient-centred communication—which captures decision-sharing language, elicitation of patient preferences, and framing of options. This is where the models have the most room for improvement.

Two caveats belong in the reading of these results. Reliability estimates for accuracy and safety were near-zero and reflect a restricted-range artefact, not genuine rater disagreement (88–92% of scores were 4 or 5); accuracy and safety comparisons should be treated as descriptive. The length-adjusted between-model and between-language comparisons on empathy, comprehensiveness, personalisation, and patient-centeredness rest on ICC(2,3) values of 0.45–0.65 and on the convergent lines of evidence detailed in the Discussion. And because only one response was generated per model–prompt combination at temperature 1.0, the model-level differences we report characterise the sampled outputs under specific API snapshots (Claude Opus 4.5, GPT-4o, Gemini 2.5 Flash, accessed 26 May 2026) at a specific point in time, not stable intrinsic properties of the underlying model families. Multi-run replication is the first-priority extension.

## Figures and Tables

**Table 1:** Response corpus by model, language, and analytic inclusion, with full word-count distributions to allow independent evaluation of alternative truncation classification schemes.

Model	Language	*n* Collected	Min	p25	Median	p75	Mean ± SD	Max	*n* < 150	*n* < 200	*n* < 250	*n* < 300	Primary Flag ^1^	Secondary Flag ^2^	Truncated (Final) ^3^	*n* Analysed
Claude Opus 4.5	EN	60	232	248	258	274	265 ± 25	355	0	0	19	56	19	52	0	60
Claude Opus 4.5	TR	60	177	220	243	273	250 ± 48	403	0	8	34	52	34	23	0	60
GPT-4o	EN	60	208	265	287	324	288 ± 38	357	0	0	8	39	8	19	0	60
GPT-4o	TR	60	116	165	193	211	190 ± 34	252	8	34	57	60	57	2	0	60
Gemini 2.5 Flash	EN	60	60	471	566	653	547 ± 150	787	2	3	4	5	4	16	0	60
Gemini 2.5 Flash	TR	60	32	58	128	231	147 ± 96	354	35	42	48	57	48	51	48 (80.0%)	12
Total		360							45	87	170	269	170	163	48 (13.3%)	312

^1^ Primary flag: word count < 250. Applied to all cells for descriptive completeness. ^2^ Secondary flag: response does not end with terminal punctuation (period, exclamation mark, question mark, closing quote, or bracket). ^3^ Truncated (final): responses classified as truncated after Stage 2 manual review (see Methods §Truncation classification for the two-stage procedure). Only Turkish Gemini responses met the Stage 2 criterion of *incomplete generation with abrupt mid-content termination*; short responses in other cells were retained as genuinely brief but complete on manual review.

**Table 2:** Post-calibration inter-rater reliability by dimension.

Dimension	ICC(2,3) [95% CI]	Krippendorff’s α (Ordinal)	Interpretation
Empathy (E)	0.56 [0.47, 0.64]	0.30	Moderate
Accuracy (D)	0.03 [0.00, 0.20]	0.01	Restricted range (see text)
Comprehensiveness (K)	0.65 [0.58, 0.72]	0.38	Moderate
Personalisation (P)	0.45 [0.34, 0.55]	0.22	Borderline moderate
Safety (G)	0.01 [0.00, 0.19]	0.01	Restricted range (see text)
Patient-centeredness (HM)	0.55 [0.45, 0.63]	0.29	Moderate
Mean	0.38	0.20	

**Table 3:** Mean (SD) scores by model, language, and dimension, with 95% confidence intervals for total scores. Sample sizes (*n* column) show the reduced Turkish Gemini cell (*n* = 12) resulting from truncation exclusion; that cell is reported descriptively only and is not entered into any inferential test.

Model	Lang	*n*	E	D	K	P	G	HM	Total [95% CI]
Claude Opus 4.5	EN	60	3.83 (0.37)	3.98 (0.21)	4.23 (0.22)	3.81 (0.28)	3.84 (0.19)	3.40 (0.27)	3.85 [3.82, 3.88]
Claude Opus 4.5	TR	60	3.21 (0.29)	3.47 (0.26)	4.07 (0.25)	3.73 (0.26)	3.75 (0.24)	3.12 (0.27)	3.56 [3.53, 3.59]
GPT-4o	EN	60	3.89 (0.29)	4.04 (0.14)	4.25 (0.28)	3.70 (0.26)	3.87 (0.16)	3.42 (0.34)	3.86 [3.83, 3.89]
GPT-4o	TR	60	3.15 (0.36)	3.66 (0.26)	3.76 (0.26)	3.58 (0.21)	3.71 (0.22)	2.94 (0.30)	3.47 [3.43, 3.50]
Gemini 2.5 Flash	EN	60	4.07 (0.34)	3.98 (0.21)	4.79 (0.36)	4.06 (0.34)	3.87 (0.21)	3.48 (0.33)	4.04 [4.00, 4.09]
Gemini 2.5 Flash	TR	12	3.24 (0.28)	3.62 (0.17)	4.10 (0.33)	3.92 (0.30)	3.74 (0.22)	3.19 (0.33)	3.64 [3.56, 3.71]

*E = Empathy, D = Accuracy, K = Comprehensiveness, P = Personalisation, G = Safety, HM = Patient-centeredness. Dimension values are mean (SD); total values are mean [95% CI]. Total-score CIs are t-distribution-based; bootstrap CIs for the n = 12 Turkish Gemini cell are provided in Supplementary S8 and are concordant with the t-based CI shown here. Extended descriptive tables (means, SDs, and reliability by dimension, plus the category × model breakdown) are provided in Supplementary S11.*

**Table 4:** Model effects for the Gemini-versus-Claude contrast on the English sample, before and after adjustment for response length. Coefficients are from mixed-effects models with scenario as a random effect; positive values indicate Gemini > Claude.

Dimension	Without Length Adjustment	With Length Adjustment
Empathy	β = +0.232 (*p* < 0.001)	β = −0.060 (*p* = 0.45)
Accuracy	β = +0.006 (*p* = 0.83)	β = +0.044 (*p* = 0.34)
Comprehensiveness	β = +0.559 (*p* < 0.001)	β = −0.073 (*p* = 0.24)
Personalisation	β = +0.256 (*p* < 0.001)	β = −0.096 (*p* = 0.11)
Safety	β = +0.028 (*p* = 0.28)	β = −0.012 (*p* = 0.78)
Patient-centeredness	β = +0.072 (*p* = 0.060)	β = −0.153 (*p* = 0.021)
Total	β = +0.192 (*p* < 0.001)	β = −0.065 (*p* = 0.017)

**Table 5:** Category-level total-score means (across the 312-response analytic set) and lowest-scoring dimension per category.

Concern Category	*n*	Total Mean	Lowest Scoring Dimension	Lowest-Scoring Dimension Mean
Aesthetics and social concerns	51	3.70	Patient-centeredness	3.27
Cost and decision-making	53	3.81	Patient-centeredness	3.53
Invasive procedures	53	3.75	Patient-centeredness	3.19
Pain and comfort	51	3.74	Patient-centeredness	3.20
Retention and long-term outcomes	51	3.75	Patient-centeredness	3.16
Treatment duration and process	53	3.74	Patient-centeredness	3.26

## Data Availability

The de-identified response corpus, scored datasets, rubric guides, prompt templates, and analysis code that support the findings of this study are available from the corresponding author on reasonable request. The data are not publicly deposited because the response corpus includes verbatim LLM outputs whose redistribution terms differ across the three commercial API providers (Anthropic, OpenAI, Google) and require case-by-case evaluation to remain compliant with each provider’s terms of service; access is provided to bona fide researchers upon request.

## Supplementary Materials

The following supporting information can be downloaded at: https://www.mdpi.com/article/10.3390/healthcare14193281/s1. The following files are provided as Supplementary Material with this submission. Items S1–S8 are compiled into a single Word document (Supplementary_S1-S8.docx) for convenience; S9 and S10 are provided as separate checklist documents; the full analysis dataset is provided as an Excel workbook (ANALYSIS_RESULTS.xlsx). S1: Uncalibrated (raw) versus calibrated main-scoring analysis: full table of dimension-level ANOVA and Tukey contrasts under both analyses. S2: Complete rubric with 5-point anchor definitions, behavioural markers, and scoring instructions distributed to raters. S3: Full 60-scenario set with English source, Turkish translation, back-translation notes, and per-scenario I-CVI values from the external orthodontist review. S4: Full English and Turkish prompt templates, verbatim, with back-translation of the Turkish version. S5: Data collection script ortho_collect_v2.py and analysis script pilot_kappa.py with commit hashes. S6: Truncation-rule sensitivity analysis: flag counts under alternative punctuation windows (40, 60, 80, 120 characters) and word-count thresholds (150, 200, 250 words). S7: Category-level descriptive breakdown of mean (SD) scores by concern category × model × language. S8: Bootstrap 95% confidence intervals (10,000 resamples) for Turkish Gemini means on the twelve non-truncated responses. S9: Completed STROBE reporting checklist for cross-sectional studies. S10: Completed TRIPOD-LLM 2025 items applicable to LLM evaluation studies. S11. Descriptive statistics tables. S11.1. Mean scores by model and language. S11.2. Standard deviations by model and language. S11.3. Inter-rater reliability by dimension. S11.4. Category × model breakdown. S12. Response length correlation, ANCOVA, and secondary truncation classifier. S12.1. Pearson correlation between response length (word count) and each rubric dimension score, computed on the full 312-response calibrated dataset. S12.2. One-way ANCOVA on the balanced English-only sample (n = 180), total score. S12.3. Secondary truncation classifier (sentence-final punctuation + recognised closing conventions). S12.4. Rating distribution for ceiling dimensions (Accuracy, Safety). S12.5. Inter-dimension correlation matrix (composite score defense). S12.6. Case-level safety detail: the three individual ‘1’ safety ratings. S13. Mixed-effects models with scenario as random effect. S13.1. Full mixed-effects output for total score, English-only sample. S13.2. Ordinal mixed-effects sensitivity: cumulative-link mixed models fitted on the raw per-rater ordinal scores (1–5 Likert) yielded coefficient signs and significance patterns concordant with the linear mixed models reported above; the length-adjustment finding (Gemini advantage eliminated after length adjustment across empathy, comprehensiveness, personalisation; reversed for total score and patient-centeredness) was reproduced.

## Abbreviations

The following abbreviations are used in this manuscript:

AI	Artificial intelligence
ANOVA	Analysis of variance
API	Application programming interface
C1	Common European Framework of Reference for Languages, level C1 (advanced)
CVI	Content validity index
D	Accuracy (dimension abbreviation, from Turkish “Doğruluk”)
E	Empathy
EN	English
FAQ	Frequently asked questions
G	Safety (dimension abbreviation, from Turkish “Güvenlik”)
HM	Patient-centeredness (dimension abbreviation, from Turkish “Hasta Merkezli”)
I-CVI	Item-level content validity index
ICC	Intraclass correlation coefficient
K	Comprehensiveness (dimension abbreviation, from Turkish “Kapsam”)
LLM	Large language model
P	Personalisation
S-CVI/Ave	Scale-level content validity index, averaging approach
SD	Standard deviation
STROBE	Strengthening the Reporting of Observational Studies in Epidemiology
TR	Turkish
TRIPOD-LLM	Transparent Reporting of a multivariable model for Individual Prognosis Or Diagnosis, for Large Language Models
