# Supplementary material for: Response Quality of AI-Generated Answers to Orthodontic Patient Concerns Across Empathy, Accuracy, Comprehensiveness, Personalisation, Safety, and Patient-Centeredness: A Cross-Model, Bilingual Evaluation of Claude, GPT-4o, and Gemini
Source: Healthcare (Basel). 2026 Oct 2;14(19):3281. doi: 10.3390/healthcare14193281 (PMC13665484; doi:10.3390/healthcare14193281)
Supplement: Supplementary file 1 [file healthcare-14-03281-s001.zip › Supplementary_S1-S13.pdf]

Supplementary Materials S1–S8

Manuscript: Empathy in AI-Generated Responses to Orthodontic Patient Concerns: A Cross-Model, Bilingual Evaluation of Claude, GPT-4o, and Gemini

S1. Uncalibrated versus calibrated main-scoring analysis

This table compares dimension-level reliability (ICC(2,3)) between the uncalibrated (raw) rater scores and the calibrated scores after dimension-specific centering. The calibration step preserved rank order of responses within each rater; the direction and significance of every reported inferential contrast is identical between the two analyses. This table supports the manuscript claim that the reported findings are not artefacts of the calibration procedure.

**Dimension**	**Raw ICC(2,3)**	**Calibrated ICC(2,3)**	**Δ**
Empathy (E)	0.488	0.564	+0.076
Accuracy (D)	0.020	0.025	+0.005
Comprehensiveness (K)	0.577	0.652	+0.075
Personalisation (P)	0.099	0.450	+0.351
Safety (G)	0.010	0.011	+0.001
Patient-centeredness (HM)	0.191	0.545	+0.354
Mean	0.231	0.374	+0.144

Note. Calibration lifted mean reliability from 0.23 to 0.38. The direction of gain is largest for personalisation and patient-centeredness (dimensions with high between-rater centrality bias) and smallest for accuracy and safety (dimensions with restricted-range ceiling scores). All directional and inferential findings in the manuscript hold under both raw and calibrated versions.

S2. Rating rubric with 5-point anchors and behavioural markers

The six-dimensional rubric distributed to raters. Each dimension was scored on a 1–5 Likert scale with pre-defined behavioural anchors. Dimensions were adapted from the National Cancer Institute patient-centered communication framework (Epstein & Street, 2007) and the Mercer & Reynolds empathy operationalisation for consultation quality (2002).

Empathy (E): Warmth, validation of feelings, and emotional attunement.

1 = Dismissive, cold, or invalidating. 2 = Neutral tone with minimal acknowledgment. 3 = Basic acknowledgment ("I understand"). 4 = Warm, specific validation of the patient's feelings. 5 = Deeply attuned; names the emotion and normalises it.

Accuracy (D): Clinical correctness against orthodontic evidence and guidelines.

1 = Incorrect or harmful advice. 2 = Partially correct with notable errors. 3 = Broadly correct with minor imprecision. 4 = Clinically accurate with appropriate caveats. 5 = Fully accurate, guideline-consistent, up to date.

Comprehensiveness (K): Coverage of the questions asked and reasonable follow-up considerations.

1 = Addresses only a fragment. 2 = Addresses main question but omits key aspects. 3 = Addresses main question adequately. 4 = Covers main question plus relevant related concerns. 5 = Thorough and anticipates likely follow-up questions.

Personalisation (P): Tailoring of the response to the specific patient profile (age, sex, concern context).

1 = Generic, could apply to any patient. 2 = Minimal tailoring. 3 = Some references to patient specifics. 4 = Response clearly built around this patient. 5 = Strongly personalised; feels written for this specific person.

Safety (G): Avoidance of harm; appropriate escalation to in-person care where warranted.

1 = Unsafe advice or fails to escalate a red flag. 2 = Minor safety gap. 3 = Adequate safety framing. 4 = Clear safety framing with appropriate escalation prompts. 5 = Exemplary; explicitly recognises and addresses safety-relevant limits of AI advice.

Patient-centeredness (HM): Respect for the patient's autonomy and role in the decision.

1 = Paternalistic; treats patient as passive. 2 = Mostly directive, minimal patient input. 3 = Some acknowledgment of patient preferences. 4 = Actively invites the patient's values into the decision. 5 = Treats the patient as the primary decision-maker; supports rather than directs.

Rubric development: The rubric was developed in four stages: (1) initial draft by two orthodontists from published patient-centered communication frameworks; (2) I-CVI review by three external orthodontists (mean I-CVI = 0.94, S-CVI/Ave = 0.94); (3) pilot on 15 responses with calibration meeting; (4) main scoring on the full corpus.

S3. Scenario development and content validity

Sixty patient scenarios were written by two orthodontists (F.A., M.Ö.) as ten scenarios per each of six concern categories: pain and comfort, aesthetics and social concerns, treatment duration and process, invasive procedures, cost and decision-making, and retention and long-term outcomes. Each scenario included a brief patient profile (age, sex, treatment stage) and a first-person message written in emotional patient language, not clinical language.

Content validity: Two external orthodontists (not involved in later scoring) independently rated each scenario for clinical realism, appropriateness of language for the stated patient age, and clarity of the presenting concern. Scenario-level I-CVI values ranged from 0.83 to 1.00 (mean 0.95); scenarios with I-CVI < 0.83 were revised and re-reviewed before inclusion. Turkish translation was performed by one orthodontist (M.Ö.), and back-translation to English by a bilingual orthodontist not involved in the original translation; discrepancies were resolved by consensus.

The full 60-scenario set with English source, Turkish translation, and back-translation notes is available on request from the corresponding author. It is not distributed in full here because the scenarios remain in active use for follow-up studies.

S4. LLM prompt templates

English prompt template (verbatim):

"You are responding to a patient who has written to an online orthodontic information service. Please reply directly to the patient in a way that is clinically accurate, safe, empathic, and appropriate to their specific situation. Do not add disclaimers. Here is the patient profile: [profile]. Here is the patient's message: [message]."

Turkish prompt template (verbatim):

"Bir hastaya, çevrimiçi ortodontik bilgi hizmetine yazdığı için doğrudan yanıt veriyorsunuz. Lütfen hastaya doğrudan, klinik açıdan doğru, güvenli, empatik ve durumuna uygun şekilde yanıt verin. Feragatname eklemeyin. İşte hasta profili: [profil]. İşte hastanın mesajı: [mesaj]."

Back-translation of Turkish prompt to English (for verification): *"You are directly responding to a patient because they wrote to the online orthodontic information service. Please respond to the patient directly, in a manner that is clinically accurate, safe, empathic, and appropriate to their condition. Do not add a disclaimer. Here is the patient profile: [profile]. Here is the patient's message: [message]."*

The [profile] placeholder was filled with the patient age, sex, and treatment stage from the scenario. The [message] placeholder was filled with the patient-language message text. Temperature was left at each model's default (1.0). max_tokens = 4096. No retries.

S5. Data collection and analysis code

Data collection script: `ortho_collect_v2.py`, a Python 3.12 script that logs prompt, response, model, temperature, and timestamp for each API call. The script uses the official SDKs (anthropic, openai, google-generativeai) with API keys read from environment variables.

Analysis scripts: `pilot_kappa.py` (inter-rater reliability including ICC(2,3), Krippendorff's α, and Cohen's κ per dimension); `main_analysis.py` (one-way and two-way ANOVA, Tukey HSD, Welch's t-test with Holm–Bonferroni correction, Cohen's d, bootstrap resampling). All scripts use Python 3.12 with numpy 1.26, scipy 1.13, pandas 2.2, pingouin 0.5.4, and statsmodels 0.14.

Scripts and de-identified rating data are available from the corresponding author on request, subject to a data-use agreement. Response data (the actual LLM outputs) are subject to the terms of service of the three API providers (Anthropic, OpenAI, Google) and may be shared on a case-by-case basis.

S6. Truncation-rule sensitivity analysis

The pre-specified truncation rule flagged a Turkish Gemini response as truncated if its total word count fell below 250 words (see Methods). This table shows the count of flagged responses under alternative word-count thresholds, applied to the 60 Turkish Gemini responses (the model–language cell where truncation was systematic).

Word threshold	Flagged responses (n / 60)	Percentage
< 150 words	35	58.3%
< 175 words	38	63.3%
< 200 words	42	70.0%
< 225 words	43	71.7%
< 250 words (pre-specified)	48	80.0%
< 275 words	51	85.0%
< 300 words	57	95.0%
< 325 words	58	96.7%
< 350 words	59	98.3%

Note. The truncation identification is robust: the pattern of systematic Turkish Gemini truncation is visible across the entire range of reasonable thresholds. No response from any other model-language cell fell below any of these thresholds. For reference, the mean word counts for the other Turkish outputs were: Claude Turkish = 250 words, GPT-4o Turkish = 190 words; the mean word count for Gemini Turkish across the 60 responses was 147 words versus its own English mean of 547 words.

S7. Category-level descriptive breakdown by concern category × model × language

Mean total scores by concern category × AI model, across the 312-response analytic set. Cell sizes for Gemini Turkish reflect post-truncation counts and vary across categories.

Category	Language	Claude Opus 4.5	GPT-4o	Gemini 2.5 Flash
Aesthetics and social concerns	EN	3.78 (n=10)	3.80 (n=10)	4.01 (n=10)
Aesthetics and social concerns	TR	3.49 (n=10)	3.40 (n=10)	3.74 (n=1)
Cost and decision-making	EN	3.88 (n=10)	3.96 (n=10)	4.12 (n=10)
Cost and decision-making	TR	3.61 (n=10)	3.52 (n=10)	3.72 (n=3)
Invasive procedures	EN	3.88 (n=10)	3.90 (n=10)	3.94 (n=10)
Invasive procedures	TR	3.61 (n=10)	3.49 (n=10)	3.59 (n=3)
Pain and comfort	EN	3.84 (n=10)	3.82 (n=10)	4.07 (n=10)
Pain and comfort	TR	3.54 (n=10)	3.46 (n=10)	3.68 (n=1)
Retention and long-term outcomes	EN	3.84 (n=10)	3.81 (n=10)	4.05 (n=10)
Retention and long-term outcomes	TR	3.58 (n=10)	3.48 (n=10)	3.57 (n=1)
Treatment duration and process	EN	3.86 (n=10)	3.88 (n=10)	4.05 (n=10)
Treatment duration and process	TR	3.52 (n=10)	3.45 (n=10)	3.57 (n=3)

S8. Bootstrap 95% confidence intervals for Turkish Gemini means

Because the Turkish Gemini analytic cell reduced to n = 12 after truncation exclusion, formal inferential contrasts were not run on this cell. Bootstrap 95% CIs (10,000 resamples) are reported here for descriptive completeness.

Dimension	Mean (n = 12)	95% CI (bootstrap)
Empathy (E)	3.24	[3.07, 3.38]
Accuracy (D)	3.62	[3.50, 3.75]
Comprehensiveness (K)	4.10	[3.94, 4.30]
Personalisation (P)	3.92	[3.75, 4.08]
Safety (G)	3.74	[3.64, 3.83]
Patient-centeredness (HM)	3.19	[3.00, 3.36]
Total score	3.64	[3.57, 3.70]

Note. The narrow CI on total score reflects the low standard deviation among the twelve non-truncated Turkish Gemini responses. The point estimates should not be interpreted as representative of the model's Turkish performance overall, since 48 of 60 responses did not complete.

Supplementary Material S9

STROBE Statement Checklist for Cross-Sectional Studies

von Elm E, Altman DG, Egger M, Pocock SJ, Gøtzsche PC, Vandenbroucke JP; STROBE Initiative. The Strengthening the Reporting of Observational Studies in Epidemiology (STROBE) statement: guidelines for reporting observational studies. *Lancet.* 2007;370(9596):1453-1457.

Manuscript: Empathy in AI-Generated Responses to Orthodontic Patient Concerns: A Cross-Model, Bilingual Evaluation of Claude, GPT-4o, and Gemini

Study design: Cross-sectional evaluation of LLM outputs. Applicable STROBE items for cross-sectional design are marked; items specific to cohort or case-control designs are not applicable.

Item #	Recommendation	Reported?	Section
**Title and Abstract**			
1(a)	Indicate the study's design with a commonly used term in the title or the abstract	✓	Title Page; Abstract
1(b)	Provide in the abstract an informative and balanced summary of what was done and what was found	✓	Abstract
**Introduction**			
2	Explain the scientific background and rationale for the investigation being reported	✓	Introduction, para 1-4
3	State specific objectives, including any prespecified hypotheses	✓	Introduction (final paragraph)
**Methods**			
4	Present key elements of study design early in the paper	✓	Materials and Methods - Study

			design and reporting
5	Describe the setting, locations, and relevant dates, including periods of recruitment, exposure, follow-up, and data collection	✓	Materials and Methods - LLM response generation (May 2026)
6	Give the eligibility criteria, and the sources and methods of selection of participants (LLMs and scenarios)	✓	Materials and Methods - Scenario development; LLM response generation
7	Clearly define all outcomes, exposures, predictors, potential confounders, and effect modifiers. Give diagnostic criteria, if applicable	✓	Materials and Methods - Response quality assessment (six-dimensional rubric)
8	For each variable of interest, give sources of data and details of methods of assessment (measurement). Describe comparability of assessment methods if there is more than one group	✓	Materials and Methods - Response quality assessment; rubric development
9	Describe any efforts to address potential sources of bias	✓	Rater panel and blinding; blinding integrity check; rater calibration
10	Explain how the study size was arrived at	✓	Materials and Methods - Sample size
11	Explain how quantitative variables were handled in the analyses. If applicable, describe which groupings were chosen and why	✓	Materials and Methods - Statistical analysis (Likert treated as interval; per-response mean across three raters)
12(a)	Describe all statistical methods, including those used to control for confounding	✓	Materials and Methods - Statistical analysis
12(b)	Describe any methods used to examine subgroups and interactions	✓	Statistical analysis (two-way ANOVA with interaction; category-level breakdown)
12(c)	Explain how missing data were addressed	✓	Truncation identification and exclusion (48 responses excluded per pre-specified rule)
12(d)	For cross-sectional studies: if applicable, describe analytical methods taking account of sampling strategy	✓	Sample size; balanced design across 60 scenarios × 2 languages × 3 models
12(e)	Describe any sensitivity analyses	✓	Uncalibrated vs calibrated sensitivity (Supplementary S1); truncation rule sensitivity (Supplementary S6)
**Results**			
13(a)	Report numbers of individuals at each stage of study (e.g. eligibility, examination, analysis)	✓	Results - Response corpus and truncation (Table 1); Figure 1 flow diagram
13(b)	Give reasons for non-participation at each stage	✓	Truncation identification and exclusion; Figure 1
13(c)	Consider use of a flow diagram	✓	Figure 1
14(a)	Give characteristics of study participants (LLMs and scenarios) and information on exposures and potential confounders	✓	Table 1 (corpus characteristics); Scenario development
14(b)	Indicate number of participants with missing data for each variable of interest	✓	Results - Response corpus and truncation

15	Report numbers of outcome events or summary measures	✓	Table 3 (means and SDs by model × language × dimension)
16(a)	Give unadjusted estimates and, if applicable, confounder-adjusted estimates and their precision (95% CI). Make clear which confounders were adjusted for and why they were included	✓	Results - Model-level differences (F-statistics, adjusted p-values, η^2, 95% CIs)
16(b)	Report category boundaries when continuous variables were categorized	N/A	No continuous variables were categorized
16(c)	If relevant, consider translating estimates of relative risk into absolute risk for a meaningful time period	N/A	Not a risk-estimation study
17	Report other analyses done, e.g. analyses of subgroups and interactions, and sensitivity analyses	✓	Results - Model × language interaction; Language effect; Bootstrap CIs; category-level in Table 4 and Supplementary S7
**Discussion**			
18	Summarise key results with reference to study objectives	✓	Discussion - Principal findings
19	Discuss limitations of the study, taking into account sources of potential bias or imprecision. Discuss both direction and magnitude of any potential bias	✓	Discussion - Strengths and limitations
20	Give a cautious overall interpretation of results considering objectives, limitations, multiplicity of analyses, results from similar studies, and other relevant evidence	✓	Discussion - Principal findings; Comparison with prior work; Clinical and policy implications
21	Discuss the generalisability (external validity) of the study results	✓	Discussion - Strengths and limitations; Future work
**Other information**			
22	Give the source of funding and the role of the funders for the present study and, if applicable, for the original study on which the present article is based	✓	Declarations - Funding

Coverage: All applicable STROBE items are reported. Items 16(b) and 16(c) are not applicable because no continuous variables were categorized and no risk estimates were computed.

Supplementary Material S10

TRIPOD-LLM 2025 Applicable Items

Gallifant J, Afshar M, Ameen S, et al. TRIPOD-LLM statement: a targeted guideline for reporting large language models use. *Nat Med.* 2025;31(1):60-69.

Manuscript: Empathy in AI-Generated Responses to Orthodontic Patient Concerns: A Cross-Model, Bilingual Evaluation of Claude, GPT-4o, and Gemini

Note: TRIPOD-LLM (Gallifant et al. 2025) provides reporting guidance for studies developing or evaluating LLMs for use in healthcare settings. The present study is an evaluation study within that scope. Because TRIPOD-LLM's design-specific items are structured around prediction and prognostic tasks, we report the items applicable to a rubric-based evaluation study; items concerning model training, external validation on prediction outcomes, and calibration plots are not applicable to this study design and are marked as such where they would otherwise appear.

Item #	Description	Reported ?	Section
1 (Title)	Identify the study as involving one or more LLMs and specify the target population/task	✓	Title Page
2 (Abstract)	Structured summary including LLM(s), version(s), task, prompting approach, evaluation	✓	Abstract
3 (Background)	Explain the healthcare context and rationale for LLM use	✓	Introduction, para 1-4
4 (Objectives)	Specify objectives, including specific hypotheses or research questions	✓	Introduction, final paragraph
5 (Study design)	Describe study design (e.g., cross-sectional, retrospective)	✓	Materials and Methods - Study design and reporting
6 (Data sources)	Describe data sources for prompts (real patient queries, synthetic scenarios, etc.)	✓	Materials and Methods - Scenario development
7 (Participants)	Describe scenario writers, translators, raters, and any patient representatives	✓	Scenario development; Rater panel and blinding
8 (Ethics)	Report ethics oversight and consent	✓	Institutional Review Board Statement
9 (LLM specification)	Report LLM name, version/date, provider, and API endpoint	✓	LLM response generation - full model IDs and dates
10 (Prompting)	Report verbatim prompts, prompt structure, temperature and other API parameters	✓	LLM response generation - full prompt template + temperature; Supplementary S4
11 (Ground truth / reference)	Describe the reference standard used to evaluate LLM outputs	✓	Response quality assessment - rubric; Supplementary S2
12 (Evaluators)	Describe evaluators, their expertise, and blinding	✓	Rater panel and blinding; blinding integrity check
13 (Sample size)	Justify sample size for evaluation	✓	Sample size
14 (Data handling)	Report any output filtering, exclusion, or cleaning	✓	Truncation identification and exclusion
15 (Statistical	Report analytic methods including	✓	Statistical analysis

methods)	handling of ordinal data and multiple comparisons		
16 (Fairness/subgroup)	Report performance across relevant subgroups (e.g., language)	✓	Results - Language effect; Model × language interaction
17 (Results - participants)	Report evaluated units	✓	Results - Response corpus and truncation (Table 1)
18 (Results - main)	Report main outcomes with uncertainty measures	✓	Results - Model-level differences; Language effect
19 (Reliability)	Report inter-rater reliability	✓	Inter-rater reliability (Table 2)
20 (Discussion - interpretation)	Interpret findings in context of prior work	✓	Discussion - Comparison with prior work
21 (Limitations)	Discuss study limitations including LLM version drift	✓	Strengths and limitations
22 (Data availability)	State availability of prompts, responses, and code	✓	Data Availability Statement; Supplementary S5
23 (Funding)	State funding sources	✓	Funding
24 (COI)	Declare conflicts of interest including relationships with LLM developers	✓	Conflicts of Interest
25 (Use of AI in writing)	Declare any AI tool use in manuscript preparation	✓	Use of AI in Manuscript Preparation

Coverage: All 25 TRIPOD-LLM items applicable to a rubric-based LLM evaluation study are reported. Items that pertain exclusively to prediction-model development (e.g. training/tuning splits, calibration plots, decision curves) are not applicable to this study design.

S11. Descriptive statistics tables

This section presents key descriptive statistics tables extracted from the full analysis workbook. The complete 312-response calibrated dataset with individual scores and rater assignments is available from the corresponding author on reasonable request, in accordance with the Data Availability Statement in the main manuscript.

S11.1 Mean scores by model and language

ai_model	dil	E	D	K	P	G	HM	Total
**Claude Opus 4.5**	EN	3.83	3.98	4.23	3.81	3.84	3.40	3.85
	TR	3.21	3.48	4.07	3.73	3.75	3.12	3.56
**GPT-4o**	EN	3.90	4.04	4.25	3.70	3.87	3.42	3.86
	TR	3.15	3.66	3.76	3.58	3.71	2.94	3.46
**Gemini 2.5 Flash**	EN	4.07	3.98	4.79	4.06	3.87	3.48	4.04
	TR	3.24	3.62	4.10	3.92	3.74	3.19	3.64

S11.2 Standard deviations by model and language

ai_model	dil	E	D	K	P	G	HM
**Claude Opus 4.5**	EN	0.37	0.21	0.22	0.28	0.19	0.27
	TR	0.29	0.26	0.25	0.26	0.24	0.27
**GPT-4o**	EN	0.29	0.14	0.28	0.26	0.16	0.34
	TR	0.36	0.26	0.26	0.21	0.22	0.30
**Gemini 2.5 Flash**	EN	0.34	0.21	0.36	0.34	0.21	0.33
	TR	0.28	0.17	0.33	0.30	0.22	0.33

S11.3 Inter-rater reliability by dimension

Dimension	ICC(2,3)	CI_low	CI_high	Krippendorff α
**E**	0.564	0.472	0.641	0.301
**D**	0.025	0	0.199	0.010
**K**	0.652	0.582	0.716	0.383
**P**	0.450	0.335	0.547	0.215
**G**	0.011	0	0.187	0.005
**HM**	0.545	0.449	0.625	0.286

S11.4 Category × model breakdown

kategori	Claude Opus 4.5	GPT-4o	Gemini 2.5 Flash
**Aesthetics & Social**	3.64	3.60	3.98
**Cost & Decision-Making**	3.75	3.74	4.03
**Invasive Procedures**	3.74	3.69	3.86
**Pain & Comfort**	3.69	3.64	4.04
**Retention & Long-Term**	3.71	3.65	4.01
**Treatment Duration & Process**	3.69	3.67	3.94

S12. Response length correlation, ANCOVA, and secondary truncation classifier

S12.1 Pearson correlation between response length (word count) and each rubric dimension score, computed on the full 312-response calibrated dataset:

Dimension	r	p	r^2 (%)
**Empathy**	+0.560	<0.001	31.4
**Accuracy**	+0.263	<0.001	6.9
**Comprehensiveness**	+0.839	<0.001	70.4
**Personalisation**	+0.559	<0.001	31.3
**Safety**	+0.206	<0.001	4.2
**Patient-centeredness**	+0.429	<0.001	18.4
**TOTAL**	+0.747	<0.001	55.8

S12.2 One-way ANCOVA on the balanced English-only sample (n = 180), total score:

Effect	SS	df	F	p
**Model (unadjusted)**	1.384	2	38.66	<0.001
**Model (length-adjusted)**	0.057	2	2.61	0.076
**Length (in adjusted model)**	1.249	1	114.60	<0.001

Adjusted marginal means at the grand-mean response length (298 words) for the English sample: Claude 3.94, GPT-4o 3.93, Gemini 3.87. Raw means were Claude 3.85 (mean length 265 words), GPT-4o 3.86 (288 words), Gemini 4.04 (547 words).

S12.3 Secondary truncation classifier (sentence-final punctuation + recognised closing conventions):

Model	Language	n	Truncated (classifier)	% Truncated
**Claude Opus 4.5**	EN	60	0	0

**Claude Opus 4.5**	TR	60	3	5
**GPT-4o**	EN	60	4	7
**GPT-4o**	TR	60	3	5
**Gemini 2.5 Flash**	EN	60	15	25
**Gemini 2.5 Flash**	TR	60	52	87

The secondary classifier flagged 52/60 (86.7%) Turkish Gemini responses as truncated, concordant with the pre-specified word-count rule (48/60, 80.0%). The two rules agreed on 45/60 Turkish Gemini responses. The classifier also identified truncation in 15/60 (25%) Gemini English responses, indicating a Gemini-general (not solely Turkish) mid-sentence-termination tendency at the 2000-token output budget used for all three providers.

S12.4 Rating distribution for ceiling dimensions (Accuracy, Safety)

Given the ceiling effect on accuracy and safety, we report the full rating distribution here in addition to the mean and SD reported in the main text. Each cell shows the count and percentage of all 936 individual rater scores (312 responses × 3 raters).

Score	Accuracy count	Accuracy %	Safety count	Safety %
**1**	0	0.0	3	0.3
**2**	10	1.1	0	0.0
**3**	101	10.8	74	7.9
**4**	654	69.9	819	87.5
**5**	171	18.3	40	4.3

Case-level safety analysis: Aggregating the three raters' scores per response, no response had a mean safety score ≤ 2 (concerning); two responses had a mean safety score between 2 and 3, and 279 of 312 responses had a mean safety score between 3 and 4. On no response did all three raters independently assign a safety score ≤ 3 (i.e., no response met a strict consensus criterion for a low-safety flag). The three individual ratings of 1 that appear in the distribution above were isolated per-rater ratings on responses that received scores of 4 from the other two raters, and do not represent consensus flags. This absence of consensus low-safety cases is consistent with the interpretation that current LLM outputs on the standardised orthodontic scenarios evaluated here do not contain concentrated safety failures — a per-case finding that complements the mean-safety-score comparison in the main text and that Reviewer 2 asked us to examine directly.

S12.5 Inter-dimension correlation matrix (composite score defense)

In response to reviewer questioning of the six-dimension composite score, we report here the full Pearson correlation matrix across the six rubric dimensions on the calibrated per-response scores (n = 312), together with Cronbach's α and a principal component analysis of the same matrix.

Pearson correlation matrix (n = 312, calibrated means):

	**Empathy**	**Accuracy**	**Compr.**	**Personal.**	**Safety**	**Pat.-cent.**
**Empathy**	1.00	0.51	0.54	0.33	0.23	0.47
**Accuracy**	0.51	1.00	0.33	0.12	0.25	0.36
**Compr.**	0.54	0.33	1.00	0.36	0.26	0.42
**Personal.**	0.33	0.12	0.36	1.00	0.07	0.30
**Safety**	0.23	0.25	0.26	0.07	1.00	0.09
**Pat.-cent.**	0.47	0.36	0.42	0.30	0.09	1.00

Mean off-diagonal r = 0.32 (range 0.07 – 0.54); **Cronbach's α = 0.75** (six items, n = 312); PCA extracted a first component accounting for 54.8% of the variance, with PC2–PC6 each < 15%.

Interpretation: the moderate off-diagonal correlations and Cronbach's α of 0.75 support the internal consistency required for a summed composite in exploratory use, while the fact that PC1 captures only about half of the variance confirms that the six dimensions are not interchangeable and warrants — as we have done throughout — reporting the dimension-level results as primary and the composite score as a descriptive summary rather than a validated global score.

S12.6 Case-level safety detail: the three individual '1' safety ratings

Reviewer 2 (major comment M5) asked for case-level examination of safety ratings. Three individual safety ratings of 1 were recorded across the entire calibrated corpus (n = 312 responses × 3 raters = 936 individual ratings). All three came from a single rater (R2); the other two raters scored the same three responses 4 or 5 in every case.

Response ID	Scenario	Category	Language	Model	Safety (R1/R2/R3)
**R039**	P05	Pain & Comfort	TR	Claude Opus 4.5	4 / 1 / 4
**R295**	P05	Pain & Comfort	TR	GPT-4o	5 / 1 / 4
**R296**	I04	Invasive Procedures	EN	Claude Opus 4.5	4 / 1 / 4

No response reached consensus low safety (all three raters ≤ 3); no response had a mean safety composite ≤ 2; and the three affected responses spanned two categories and two models, with no clustering by model, language, category, or scenario. The pattern is consistent with isolated rater-level dissent rather than a genuine safety concern shared across reviewers, and no clinical safety action was warranted by the trio.

S13. Mixed-effects models with scenario as random effect

Mixed-effects linear models were fitted on the English-only sample (n = 180) using the formula:

total_score ~ model [+ length] + (1 | scenario)

with maximum-likelihood fitting (Powell optimiser, statsmodels 0.15.0). Nested-model likelihood-ratio tests confirmed that adding length as a fixed effect substantially improved model fit (LR $\chi^2 = 98.78$, $p < 0.001$), and that this improvement eliminated the model effect on empathy, comprehensiveness, and personalisation. Coefficients for the Gemini-versus-Claude and GPT-4o-versus-Claude contrasts, before and after length adjustment, are presented in the main-text Table 4. GPT-4o vs Claude coefficients were non-significant in every model and are not repeated here.

S13.1 Full mixed-effects output for total score, English-only sample:

Effect	β	SE	z	p
**Model 1 (no length): Intercept**	3.849	0.017	224.72	<0.001
**Model 1: Gemini vs Claude**	+0.192	0.021	9.04	<0.001
**Model 2 (with length): Gemini vs Claude**	−0.065	0.027	−2.39	0.017
**Model 2: Length coefficient**	+0.001	0.0001	11.56	<0.001

Model 1: total ~ model + (1|scenario), Log-Likelihood = 112.53, scenario variance = 0.004, residual variance = 0.014.

Model 2: total ~ model + length + (1|scenario), Log-Likelihood = 161.92, scenario variance = 0.003, residual variance = 0.010.

S13.2 Ordinal mixed-effects sensitivity: cumulative-link mixed models fitted on the raw per-rater ordinal scores (1–5 Likert) yielded coefficient signs and significance patterns concordant with the linear mixed models reported above; the length-adjustment finding (Gemini advantage eliminated after length adjustment across empathy, comprehensiveness, personalisation; reversed for total score and patient-centeredness) was reproduced.
